# UHPLC-QQQ-MS/MS assay for the quantification of dianthrones as potential toxic markers of *Polygonum multiflorum* Thunb: applications for the standardization of traditional Chinese medicines (TCMs) with endogenous toxicity

**DOI:** 10.1186/s13020-021-00463-w

**Published:** 2021-07-03

**Authors:** Jian-Bo Yang, Yun-Fei Song, Yue Liu, Hui-Yu Gao, Qi Wang, Ying Wang, Xian-Long Cheng, Tian-Tian Zuo, Xiao-Wen Hu, Feng Wei, Hong-Tao Jin, Shu-Ting Wang, Shuang-Cheng Ma

**Affiliations:** 1 Institute for Control of Chinese Traditional Medicine and Ethnic Medicine, National Institutes for Food and Drug Control, Beijing, 100050 China; 2 School of Chinese Materia Medica, Beijing University of Chinese Medicine, Beijing, 102488 China; 3 Chinese Academy of Medical Sciences and Peking Union Medical College, Beijing, 100050 China

**Keywords:** *Polygonum multiflorum* Thunb, Dianthrones, Endogenous toxic TCMs, Toxic markers, HepaRG cells

## Abstract

**Background:**

The raw and processed roots of *Polygonum multiflorum* Thunb (PM) are commonly used in clinical practice to treat diverse diseases; however, reports of hepatotoxicity induced by Polygoni Multiflori Radix (PMR) and Polygoni Multiflori Radix Praeparata (PMRP) have emerged worldwide. Thus, it is necessary for researchers to explore methods to improve quality standards to ensure their quality and treatment effects.

**Methods:**

In the present study, an ultra-high performance liquid chromatography triple quadrupole mass spectrometry (UHPLC-QQQ-MS/MS) method was optimized and validated for the determination of dianthrones in PMR and PMRP using bianthronyl as the internal standard. Chromatographic separation with a gradient mobile phase [A: acetonitrile and B: water containing 0.1% formic acid (v/v)] at a flow rate of 0.25 mL/min was achieved on an Agilent ZORBAX SB-C_18_ column (2.1 mm × 50 mm, 1.8 μm). The triple quadrupole mass spectrometer (TQMS) was operated in negative ionization mode with multiple reaction monitoring for the quantitative analysis of six dianthrones. Moreover, compounds **5** and **6** were further evaluated for their cytotoxicity in HepaRG cells by CCK-8 assay.

**Results:**

The UHPLC-QQQ-MS/MS method was first developed to simultaneously determine six dianthrones in PMR and PMRP, namely, polygonumnolides C1**–**C4 (**1–4**), *trans*-emodin dianthrones (**5**), and *cis*-emodin dianthrones (**6**). The contents of **1**–**6** in 90 batches of PMR were in the ranges of 0.027–19.04, 0.022–13.86, 0.073–15.53, 0.034–23.35, 0.38–83.67 and 0.29–67.00 µg/g, respectively. The contents of **1**–**6** in 86 batches of commercial PMRP were in the ranges of 0.020–13.03, 0.051–8.94, 0.022–7.23, 0.030–12.75, 0.098–28.54 and 0.14–27.79 µg/g, respectively. Compounds **1**–**4** were almost completely eliminated after reasonable processing for 24 h and the contents of compounds **5** and **6** significantly decreased. Additionally, compounds **5** and **6** showed inhibitory activity in HepaRG cells with IC_50_ values of 10.98 and 15.45 μM, respectively. Furthermore, a systematic five-step strategy to standardize TCMs with endogenous toxicity was proposed for the first time, which involved the establishment of determination methods, the identification of potentially toxic markers, the standardization of processing methods, the development of limit standards and a risk–benefit assessment.

**Conclusion:**

The results of the cytotoxicity evaluation of the dianthrones indicated that *trans*-emodin dianthrones (**5**) and *cis*-emodin dianthrones (**6**) could be selected as toxic markers of PMRP. Taking PMR and PMRP as examples, we hope this study provides insight into the standardization and internationalization of endogenous toxic TCMs, with the main purpose of improving public health by scientifically using TCMs to treat diverse complex diseases in the future.

## Introduction

*Polygonum multiflorum* Thunb, including Polygoni Multiflori Radix (PMR) and PMR Praeparata (PMRP), is a commonly used TCMs used to treat various diseases in China and is also popular in many other countries [1, 2]. PMR has many common indications, including detoxification, elimination of carbuncles, malaria prevention, and relaxation of the bowel, while PMRP is well known as a tonic medicine for blackening of the hair, nourishing the liver and kidney, haematopoiesis, and so on [3–5]. However, since the 1990s, a significant number of adverse hepatotoxic reactions have occurred in China, South Korea, Japan, England, Canada, and other countries from the use of these medicines [6–8]. The chemical composition of PMR can be significantly altered by processing, and its hepatotoxicity can be minimized accordingly. Some studies have shown that processing could result in a decrease in certain compounds, such as 2,3,5,4′-tetrahydroxystilbene-2-*O*-*β-*d-glucopyranoside (THSG), emodin-8-*O*-*β-*d-glucoside, catechin, epicatechin, and physcion-8-*O*-*β-*d-glucopyranoside; however, these compounds did not disappear [2, 9, 10]. These studies demonstrated that there may be no direct link between the above mentioned compounds and PMR-induced liver injury.

Our previous work on PMR toxicity showed that the dianthrones that were first isolated from PMR by our team could have potential hepatotoxicity, and there are many minor dianthrones in PMR [11–17]. Moreover, dianthrones can increase the content of Fe^3+^ ions and degrade easily when heated [18, 19]. These features are very similar to those of the hepatotoxic components of PMR [20, 21]. However, to the best of our knowledge, there have been no reports on which types of dianthrones are toxicity markers of PMRP or the mechanisms to decrease the toxicity of these TCMs. Therefore, in this study, an effective and sensitive UHPLC-QQQ-MS/MS method was established, and the qualitative analysis of six dianthrones was presented. The excellent selectivity and sensitivity achieved for these target compounds in multi-reaction monitoring (MRM) mode allowed for satisfactory confirmation and quantitation [22]. In addition, the proposed UHPLC-QQQ-MS/MS method was successfully used for dianthrone determination in PMR and PMRP. To the best of our knowledge, this work is the most comprehensive study on the contents of dianthrones in PMR and PMRP. The results showed that there is a strong correlation between dianthrones and PMR-induced liver damage, and *trans*-emodin dianthrones (**5**) and *cis*-emodin dianthrones (**6**) could be chosen as potential toxicity markers of PMRP. Furthermore, a systematic five-step strategy to standardize TCMs with endogenous toxicity was proposed for the first time, which involved the establishment of determination methods, the identification of toxic markers, the standardization of the processing method, the development of limit standards and a risk–benefit assessment. Taking PMR as an example, it is hoped that these findings will improve the standardization and internationalization of endogenous toxic TCMs and provide indispensable evidence for ensuring safe and effective clinical treatment in the future.

In the past several decades, many human liver cell lines have been used for in vitro screening tests to evaluate hepatotoxic drugs and other compounds. The HepaRG cell line has been proven to be suitable human hepatocytes for the assessment of hepatotoxicity in vitro [23]. HepaRG cells were identified from a human hepatocellular carcinoma cell line infected with the hepatitis B virus and isolated for the first time from non-neoplastic tissue in women with chronic hepatitis C virus infection [24]. HepaRG cells are derived from highly proliferating progenitor cells, which differentiate into both biliary and hepatocellular cells in 2% dimethyl sulfoxide (DMSO) [25]. Compared with HepG2 cells and the others, HepaRG cells, which are similar to human primary hepatocytes, are capable of expressing phase I drug metabolic CYP enzymes, phase II drug metabolic enzymes, transporters, and the nuclear receptor specificity of liver functions [26]. Therefore, in this study, HepaRG cells were selected to evaluate the toxicity of *trans*-emodin dianthrones (**5**), and *cis*-emodin dianthrones (**6**) to hepatocytes in vitro.

## Materials and methods

### Reagents and materials

HPLC-grade acetonitrile was purchased from Fisher Scientific (Fair Lawn, NJ, USA). Formic acid was purchased from Merck Inc. (Darmstadt, Germany). Ethanol was of analytical grade and purchased from Shanghai Chemical Reagent Co. (Shanghai, China). Water was purified with a Milli-Q water purification apparatus (Millipore, Billerica, MA, USA). The immortalized hepatic cell line HepaRG was obtained from the Type Culture Collection of the Chinese Academy of Sciences (Shanghai, China). The following reagents were also used in this study: RPMI 1640 culture medium (Biological Industries, Israel), foetal bovine serum (Biosera, France), penicillin (Targetmol, China), staurosporine (STSP; Targetmol, China), 0.25% trypsin–EDTA (Wisent, Canada), CCK-8 reagent (Targetmol, China), DMSO (Sinopharm, China), and a Victor Nivo multi-mode plate reader (PerkinElmer, China).

*Polygonum multiflorum* samples were authenticated by Associate Professors Ji Zhang and Jian-Bo Yang (Research and Inspection Center of TCM and Ethnomedicine, National Institutes for Food and Drug Control, State Food and Drug Administration) in accordance with the Chinese Pharmacopoeia (edition 2015, volume 1) [3]. A voucher sample of PMR (No. 20191001) was collected from Deqing County, Guangdong Province, China and deposited at the TCM and Ethnomedicine Research and Inspection Center, National Institutes for Food and Drug Control, State Food and Drug Administration, Beijing, China.

**Fig. 1:**
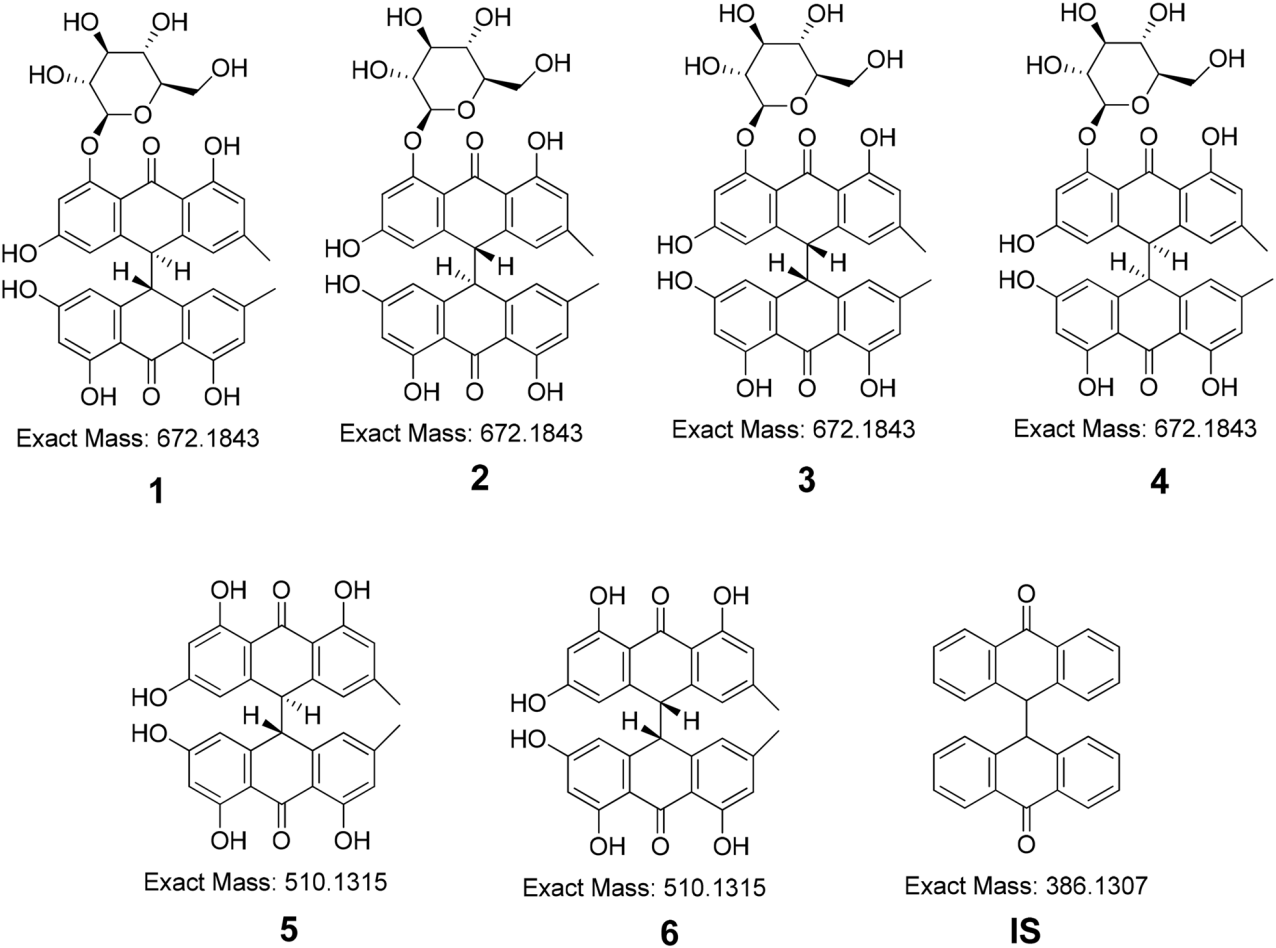
Chemical structure of six dianthrones (**1**–**6**) and one internal standard (**IS**)

The chemical compounds polygonumnolide C4 (**1**), polygonumnolide C3 (**2**), polygonumnolide C1 (**3**), polygonumnolide C2 (**4**), *trans*-emodin dianthrones (**5**), and *cis*-emodin dianthrones (**6**) were isolated and purified. The structures of the six dianthrones (**1**–**6**) were confirmed by UV, MS, ^1^H NMR and ^13^C NMR analyses, which have been reported in the literature [13–15]. The purity of these compounds was greater than 98.0% (as determined by HPLC). The internal standard (IS) (Bianthronyl) was purchased from Moving Your Chemistry Forward (Shanghai, China). Figure 1 shows the structures of the six dianthrones and one IS. All solvents and samples were filtered through 0.22 μm filters before UHPLC injection.

### Apparatus

The UHPLC-MS/MS instrument consisted of an Agilent 1200 series UHPLC system equipped with an Agilent 6410B TQMS/MS system (Agilent Technologies, Santa Clara, CA, USA). Chromatographic analyses were performed using an Agilent 1200 series UHPLC system (Agilent Technologies, Santa Clara, CA, USA) consisting of a quaternary pump, an online degasser, an auto plate-sampler, and a thermostatically controlled column compartment. Chromatographic separation was carried out at 30 °C on an Agilent ZORBAX SB-C_18_ column (2.1 mm × 50 mm, 1.8 μm). Separation was achieved with a gradient of mobile phases consisting of acetonitrile (A) and water containing 0.1% formic acid (v/v) (B) at a flow rate of 0.25 mL/min. The gradient was programmed as follows: 0–8 min, maintenance at 37% A; 8–10 min, linear change to 60% A; 10–12 min, linear change to 78% A; 12–20 min, linear change to 90% A; 20–22 min, linear change to 37% A; and 22–30 min, maintenance at 37% A. The column temperature was maintained at 30 °C. The injection volume was 2.0 μL.

All MS experiments were conducted using an ESI source in negative ion electrospray mode with a 6410B TQMS (Agilent, USA). The optimal MS conditions were as follows: drying gas temperature, 300 °C; drying gas flow rate, 10 L/min; nebulizer gas pressure, 30 psi; sheath gas temperature, 300 °C; sheath gas flow, 11 L/min and capillary voltage, 4.0 kV. Detection was carried out in MRM mode. All data were processed using MassHunter Workstation software (V.7.0 Quantitative Analysis; Agilent, USA).

### Preparation of standard solutions

Standard stock solutions for the six dianthrones, namely, polygonumnolide C4 (**1**), polygonumnolide C3 (**2**), polygonumnolide C1 (**3**), polygonumnolide C2 (**4**), *trans*-emodin dianthrones (**5**) and *cis*-emodin dianthrones (**6**), were prepared in 70% ethanol. Accordingly, a standard mixture solution was obtained by precisely mixing the six stock solutions with 70% ethanol so that the concentrations were 0.210 (**1**), 0.214 (**2**), 0.283 (**3**), 0.280 (**4**), 0.318 (**5**) and 0.280 (**6**) μg/mL. The mixture solutions were further diluted to generate standard solutions in different concentration ranges. The calibration curves were generated with at least six appropriate concentrations. Bianthronyl (**IS**) was prepared in DMSO/methanol (v/v, 2:1) at a concentration of 100.44 μg/mL. All standards solutions were stored at 4 °C.

### Sample preparation

#### Polygoni multiflori radix (PMR)

Ninety batches of PMR (PMR-01–PMR-90) were collected from different provinces of China, as shown in Table 1.

**Table 1:** Sample collection information in the present study

Samples	Location	Samples	Location
PMR-01	Bozhou, Anhui Province, China	PMRP-01	Changsha, Hunan Province, China
PMR-02	Dingxi, Gansu Province, China	PMRP-02	Bozhou, Anhui Province, China
PMR-03	Beijing, China	PMRP-03	Lijiang,Guangxi Zhuang Autonomous Region, China
PMR-04	Qujing, Yunnan Province	PMRP-04	Anguo, Heibei Province, China
PMR-05	Bozhou, Anhui Province, China	PMRP-05	Dunhua, Jilin Province, China
PMR-06	Xianyang, Shanxi Province, China	PMRP-06	Tongchuan, Shanxi Province, China
PMR-07	Bozhou, Anhui Province, China	PMRP-07	Huanggang, Hubei Province, China
PMR-08	Taizhou, Jiangsu Province, China	PMRP-08	Bozhou, Anhui Province, China
PMR-09	Tianshui, Gansu Province, China	PMRP-09	Zhangshu, Jiangxi Province, China
PMR-10	Bozhou, Anhui Province, China	PMRP-10	Chengduo, Sichuan Province, China
PMR-11	Bozhou, Anhui Province, China	PMRP-11	Shijiazhuang, Hebei Province, China
PMR-12	Zunyi, Guizhou Province, China	PMRP-12	Beijing, China
PMR-13	Sichuan Province, China	PMRP-13	Guigang, Guangxi Zhuang Autonomous Region, China
PMR-14	Shijiazhuang, Hebei Province, China	PMRP-14	Shiyan,Hubei Province, China
PMR-15	Longde, Ningxia Hui Autonomous Region, China	PMRP-15	Zhangshu, Jiangxi Province, China
PMR-16	Puyang, Anhui Province, China	PMRP-16	Quzhou, Zhejiang Province, China
PMR-17	Yuncheng, Shanxi Province, China	PMRP-17	Huzhou, Zhejiang Province, China
PMR-18	Anguo, Heibei Province, China	PMRP-18	Nanjing, Jiangsu Province, China
PMR-19	Haikou, Hainan Province, China	PMRP-19	Kunming, Yunnan Province, China
PMR-20	Xining, Qinghai Province, China	PMRP-20	Anguo, Hebei Province, China
PMR-21	Bozhou, Anhui Province, China	PMRP-21	Bozhou, Anhui Province, China
PMR-22	Shangrao, Jiangxi Province, China	PMRP-22	Beijing, China
PMR-23	Yulin, Guangxi Zhuang Autonomous Region, China	PMRP-23	Luoyang, Henan Province, China
PMR-24	Chengdou, Sichuan Province, China	PMRP-24	Kunming, Yunnan Province, China
PMR-25	Shanghai, China	PMRP-25	Chengduo, Sichuan Province, China
PMR-26	Anguo, Heibei Province, China	PMRP-26	Bozhou, Anhui Province, China
PMR-27	Jining, Shandong Province, China	PMRP-27	Heze, Shandong Province, China
PMR-28	Anguo, Heibei Province, China	PMRP-28	Bozhou, Anhui Province, China
PMR-29	Linzhi, Tibet Province, China	PMRP-29	Nantong, Jiangsu Province, China
PMR-30	Zhongxiang, Hubei Province, China	PMRP-30	Shanghai, China
PMR-31	Anguo, Heibei Province, China	PMRP-31	Zhanjiang, Guangdong Province, China
PMR-32	Chengdou, Sichuan Province, China	PMRP-32	Bozhou, Anhui Province, China
PMR-33	Shanghai, China	PMRP-33	Hangzhou, Zhejiang Province, China
PMR-34	Anguo, Heibei Province, China	PMRP-34	Chongqing, China
PMR-35	Bozhou, Anhui Province, China	PMRP-35	Guyuan, Ningxia Hui Autonomous Region, China
PMR-36	Anguo, Heibei Province, China	PMRP-36	Bozhou, Anhui Province, China
PMR-37	Yuncheng, Shanxi Province, China	PMRP-37	Bozhou, Anhui Province, China
PMR-38	Yangzhong, Jiangsu Province, China	PMRP-38	Ningbo, Zhejiang Province, China
PMR-39	Baoji, Shanxi Province, China	PMRP-39	Xian, Shanxi Province, China
PMR-40	Anguo, Heibei Province, China	PMRP-40	Yulin, Guangxi Zhuang Autonomous Region, China
PMR-41	Xichang, Jiangxi Province, China	PMRP-41	Bozhou, Anhui Province, China
PMR-42	Kunming, Yunnan Province, China	PMRP-42	Beijing, China
PMR-43	Shaoxing, Zhejiang Province, China	PMRP-43	Tianjin, China
PMR-44	Chengdou, Sichuan Province, China	PMRP-44	Anguo, Hebei Province, China
PMR-45	Chengdou, Sichuan Province, China	PMRP-45	Tianjin, China
PMR-46	Shangrao, Jiangxi Province, China	PMRP-46	Bozhou, Anhui Province, China
PMR-47	Zhongxiang, Hubei Province, China	PMRP-47	Beijing, China
PMR-48	Deqing, Guangdong Province, China	PMRP-48	Nanjing, Jiangsu Province, China
PMR-49	Deqing, Guangdong Province, China	PMRP-49	Anguo, Hebei Province, China
PMR-50	Deqing, Guangdong Province, China	PMRP-50	Yunfu, Guangdong Province, China
PMR-51	Deqing, Guangdong Province, China	PMRP-51	Bozhou, Anhui Province, China
PMR-52	Deqing, Guangdong Province, China	PMRP-52	Anguo, Hebei Province, China
PMR-53	Urumqi, Xinjiang Uygur Autonomous Region, China	PMRP-53	Xinyu, Jiangxi Province, China
PMR-54	Bozhou, Anhui Province, China	PMRP-54	Anguo, Hebei Province, China
PMR-55	Guangxi Zhuang Autonomous Region, China	PMRP-55	Qiqihaer, Heilongjiang Province, China
PMR-56	Yunnan Province, China	PMRP-56	Nanjing, Jiangsu Province, China
PMR-57	Dengfeng, Henan Province, China	PMRP-57	Haerbing, Heilongjiang Province, China
PMR-58	Bozhou, Anhui Province, China	PMRP-58	Guiyang, Guizhou Province, China
PMR-59	Bozhou, Anhui Province, China	PMRP-59	Bozhou, Anhui Province, China
PMR-60	Bozhou, Anhui Province, China	PMRP-60	Yuechang, Guangdong Province, China
PMR-61	Yunnan Province, China	PMRP-61	Chengduo, Sichuan Province, China
PMR-62	Puer, Yunan Province, China	PMRP-62	Bozhou, Anhui Province, China
PMR-63	Guizhou Province, China	PMRP-63	Beijing, China
PMR-64	Guizhou Province, China	PMRP-64	Bozhou, Anhui Province, China
PMR-65	Henan Province, China	PMRP-65	Unkonwn, China
PMR-66	Bozhou, Anhui Province, China	PMRP-66	Unkonwn, China
PMR-67	Kaili, Guizhou Province, China	PMRP-67	Unkonwn, China
PMR-68	Congjiang, GuizhouProvince, China	PMRP-68	Unkonwn, China
PMR-69	Chengdou, Sichuan Province, China	PMRP-69	Chengduo, Sichuan Province, China
PMR-70	Bozhou, Anhui Province, China	PMRP-70	Bozhou, Anhui Province, China
PMR-71	Yuzhou, Henan Province, China	PMRP-71	Puer, Yunnan Province, China
PMR-72	Henan Province, China	PMRP-72	Xichang,Sichuan Province, China
PMR-73	Sichuan Province, China	PMRP-73	Bozhou, Anhui Province, China
PMR-74	Bozhou, Anhui Province, China	PMRP-74	Bozhou, Anhui Province, China
PMR-75	Hengyang, Hunan Province, China	PMRP-75	Chengduo, Sichuan Province, China
PMR-76	Deqing, Guangdong Province, China	PMRP-76	Bozhou, Anhui Province, China
PMR-77	Deqing, Guangdong Province, China	PMRP-77	Lijiang, Yunnan Province, China
PMR-78	Henan Province, China (2018 year)	PMRP-78	Deqing, Guangdong Province, China
PMR-79	Sichuan Province, China	PMRP-79	Honghe, Yunnan Province, China
PMR-80	Deqing, Guangdong Province, China	PMRP-80	Henan Province, China
PMR-81	Deqing, Guangdong Province, China	PMRP-81	Henan Province, China
PMR-82	Guizhou Province, China	PMRP-82	Sichuan Province, China
PMR-83	Guizhou Province, China	PMRP-83	Sichuan Province, China
PMR-84	Sichuan Province, China	PMRP-84	Sichuan Province, China
PMR-85	Dengfeng, Henan Province, China	PMRP-85	Henan Province, China
PMR-86	Sichuan Province, China	PMRP-86	Henan Province, China
PMR-87	Bozhou, Anhui Province, China		
PMR-88	Lijiang, Yunnna Province, China		
PMR-89	Guangxi Zhuang Autonomous Region, China		
PMR-90	Guizhou Province, China		

#### Polygoni Multiflori Radix Praeparata (PMRP)

PMRP can improve the efficacy and reduce the hepatotoxicity of PMRP after processing. PMRP could be extracted from PMR using the method from the Chinese Pharmacopoeia (2020 edition) [3] and traditional methods [27]. Eighty-six batches of PMR (PMRP-01–PMRP-86) were collected from different provinces of China, as shown in Table 1.

The water-steaming method was as follows: A sample (PMR-49) was collected for examination at different points and labelled PMRP-S_0h_, S_2h_, S_4h_, S_6h_, S_8h_, S_10h_, S_12h_, S_16h_, S_20h,_ or S_24h_. In addition, ten samples of PMRP-(S_0h_–S_24h_) were successfully obtained. Moreover, 15 batches of crude PMR (300 g) were infiltrated by distilled water and steamed at 100 °C for 0, 12, or 24 h. These processed products were then dried in the sunlight. Finally, 45 samples of PMRP-(SZ01-0h, SZ01-12h, and SZ01-24h and SZ15-0h, SZ15-12h, and SZ15-24h) were successfully obtained.

### Sample analysis

An aliquot of 1.0 g of PMR or PMRP (filtered through a no. 3 sieve) was weighed into a stoppered conical flask, 50 mL of accurately measured ethanol–water (7:3, v/v) was added followed by weighing and ultrasonication (power, 100 W; frequency, 40 kHz) for 30 min. The solution was cooled and weighed again, the loss of weight was replenished with ethanol–water (7:3, v/v) and the solution was mixed well. This extract was then filtered through a 0.22 μm syringe filter. The filtrate was used as the test solution and analysed with UHPLC-QQQ-MS/MS according to the above procedure.

### Cytotoxic effects of dianthrone exposure in HepaRG cells

HepaRG cells were maintained in RPMI 1640 medium containing 10% FBS, 100 U/mL penicillin and streptomycin at 37 °C with 5% CO_2_. The effects of the toxic dianthrone markers on HepaRG cell viability were determined using a CCK-8 assay. According to the experimental operation requirements, the day before detection, HepaRG cells were inoculated in 384-well cell plates at a density of 1000 cells/well with 40 μL of cell suspension inoculated in each well. The cell plates were placed in an incubator at 37 °C with 5% CO_2_ for overnight incubation. On the day of the experiment, 10 μL of compound working solution (0.064, 0.32, 1.6, 8, 40, 200 or 1000 μg/mL) was added to each well, and the plate was incubated at 37 °C in a 5% CO_2_ incubator in the dark for 72 h. After incubation, 5 μL of CCK-8 reagent was added to each cell well followed by incubation for 4 h. The absorbance at 450 nm was measured on the NIVO instrument, and the inhibition rate was calculated by the following formula:

$${\text{Inhibition}}\;{\text{ratio}}\left( \% \right) = \left( {{\text{Ods}}\, - \,{\text{OD}}_{{{\text{NC}}}} } \right)/\left( {{\text{OD}}_{{{\text{STSP}}}} \, - \,{\text{OD}}_{{{\text{NC}}}} } \right)\, \times \,100\%$$

where Ods is the absorbance of the sample solution (cell + medium + compound to be tested), OD_NC_ is the absorbance of the negative control (cell + medium + DMSO), and OD_STSP_ is the absorbance of the positive control (cell + medium + 10 μM STSP). According to the inhibition ratios of the compounds, the IC_50_ values (the concentration corresponding to 50% of the maximum inhibition response) were calculated from the dose–response curves using GraphPad. All tests were conducted in triplicate, and the mean values were finally obtained.

## Results and discussion

### Optimization of the extraction method

**Fig. 2:**
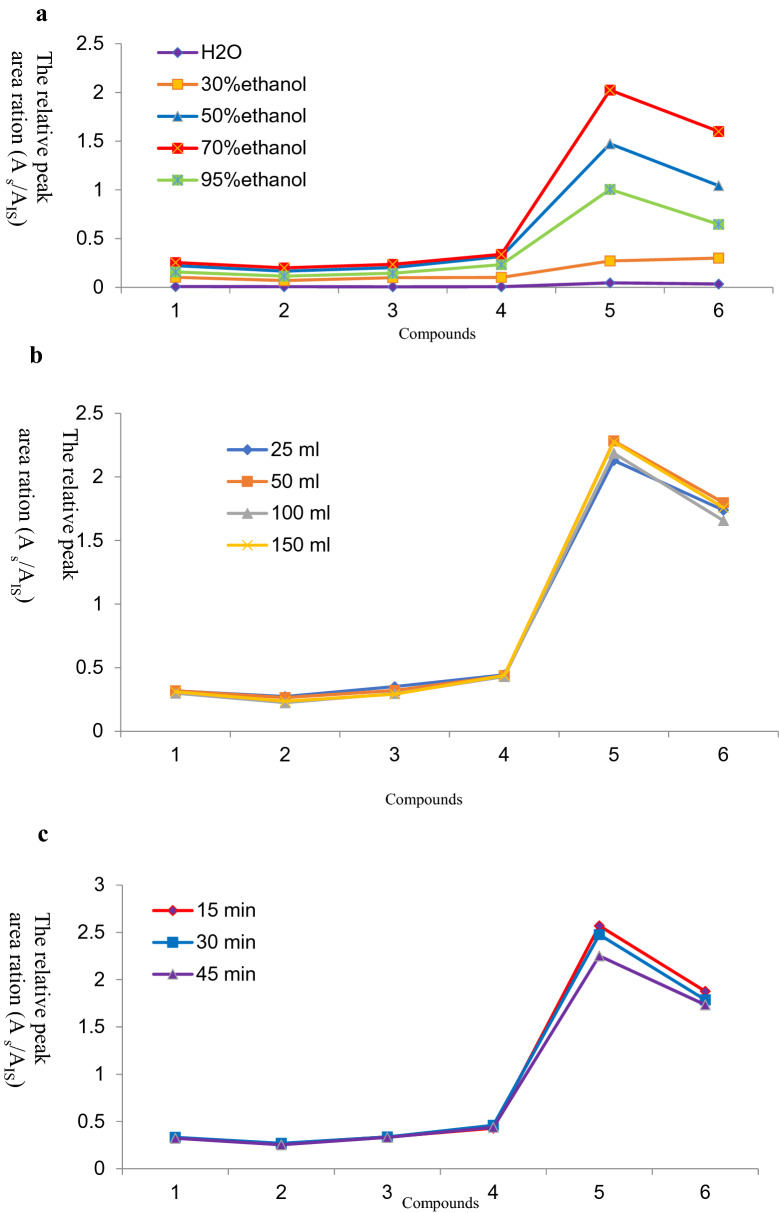
Optimization of different parameters of the method of sample solution, **A** type of extractant, **B** volume of extractant and **C** ultrasound time

PMR (No. 20191001) was used to optimize the extraction process. Optimization was successfully completed using a three-step approach, described as follows. Step 1. *Optimization of the extraction solvent system*: the first step in the preparation of the sample solution was to select a suitable extraction solvent because of its paramount role in achieving good recovery. Five solutions, H_2_O and 30%, 50%, 70%, and 95% ethanol (v/v in water), were systematically compared by virtue of the peak areas of the six dianthrones in PMR. As a result, 70% ethanol exhibited the highest extraction efficiency among the tested solvents, as shown in Fig. 2A. Hence, 70% aqueous ethanol was selected as the best extraction solvent for this study. Step 2. *Optimization of solvent volume*: extractant volume may be another factor that could affect extraction efficiency. This study aimed to obtain the minimum volume of extractant required to achieve the highest extraction efficiency. Four different volumes of 70% ethanol (25, 50, 100 and 150 mL) were systematically studied. From Fig. 2B, the peak areas of the six dianthrones increased with increasing volume of 70% ethanol. However, there was no significant difference among the results of four different volumes of 70% ethanol. Therefore, 50 mL of 70% ethanol was eventually selected as the optimized volume for environmentally friendly reasons. Step 3. *Optimization of ultrasonication time*: in this study, an ultrasonic process was used to extract the six dianthrones from PMR. From Fig. 2C, there was no significant difference among ultrasonication times of 15, 30, and 45 min. Accordingly, 30 min was selected as the best extraction time to save energy.

In conclusion, the optimal sample preparation method was found to be the extraction of a 1.0 g sample with 50 mL of 70% ethanol in an ultrasonic water bath for 30 min.

### Optimization of UHPLC-QQQ-MS/MS conditions

**Fig. 3:**
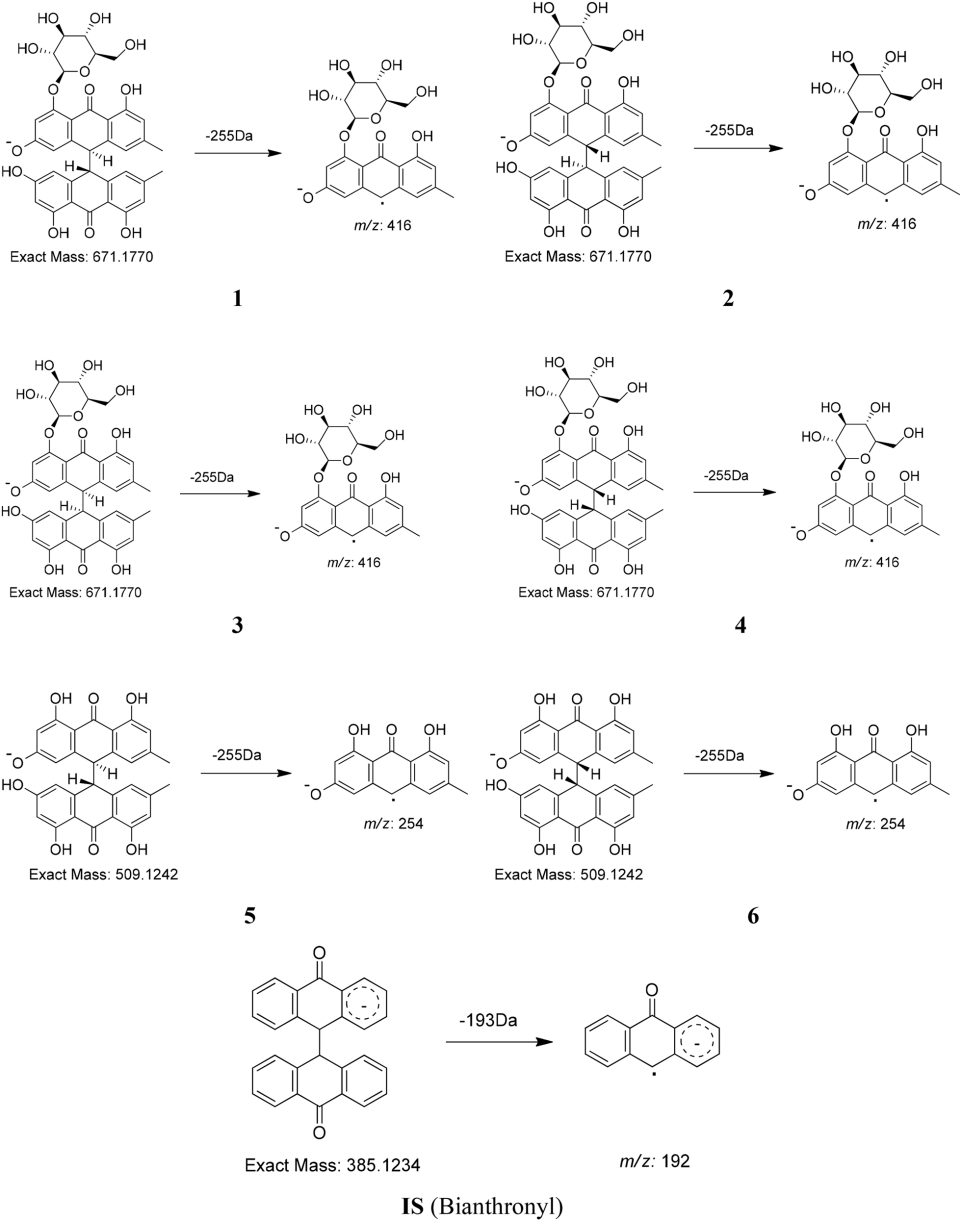
Product ion mass spectra and proposed fragmentation pathway of six dianthrones (**1**–**6**) and one internal standard (**IS**)

The chromatographic conditions, especially the composition of the mobile phase, were optimized to achieve the best possible resolution and symmetric peaks of the seven compounds within a suitable run time. Over the course of the tests, four mobile phases were examined, i.e., methanol-, acetonitrile-, and methanol–water containing 0.1% formic acid (v/v), and acetonitrile–water containing 0.1% formic acid (v/v), in different ratios. The acetonitrile–water containing 0.1% formic acid (v/v) combination had the lowest pressure, best baseline stability, and highest ionization efficiency among those tested and was eventually selected as the mobile phase. Both positive and negative ion modes were also tested for MS analysis. The seven compounds showed cleaner mass spectral backgrounds and higher sensitivities in negative mode than in positive mode. The parameters of fragmented voltage and collision energy were optimized to obtain the richest relative abundance of parent ions and outputs for the optimization of MRM conditions. In addition, the MRM transitions and parameters of these seven dianthrone compounds are shown in Table 2. Other parameters, such as dry gas flow rate, temperature, nebulizer, and capillary voltage were set to 10.0 L/min, 300 °C, 15 psi, and 4000 V, respectively. The production mass spectra and proposed fragmentation pathways of **1**–**6** and the IS are also shown in Fig. 3. These seven dianthrones (**1**–**6** and **IS**) identify cleavage of the C10–C10′ bond to yield anthrone-free radicals in the MS/MS product ion spectra. The MS/MS product ion spectra of **1**–**6** were reported in the article [12].

**Table 2:** Parameters of dianthrones of 6 analytes and 1 internal standard in MRM analysis

No.	Compounds	Retention times (RT, min)	Precursor ion (*m/z*)	Product ion (*m/z*)	Fragmentor voltage (FV)	Collision energy (CE)	Ion mode
1	Polygonumnolide C4	6.99	670.7 [M−H]^−^	415.8	100	25	(−) ESI
2	Polygonumnolide C3	7.63	671.0 [M−H]^−^	415.8	100	25	(−) ESI
3	Polygonumnolide C1	11.80	670.9 [M−H]^−^	415.9	100	25	(−) ESI
4	Polygonumnolide C2	13.25	670.9 [M−H]^−^	416.0	100	25	(−) ESI
5	*trans*-Emodin dianthrones	16.51	508.8 [M−H]^−^	253.8	150	25	(−) ESI
6	*cis*-Emodin dianthrones	17.06	508.7 [M−H]^−^	253.9	150	25	(−) ESI
1S	Bianthronyl	17.37	384.9 [M−H]^−^	191.8	100	30	(−) ESI

### Method validation

#### Specificity

**Fig. 4:**
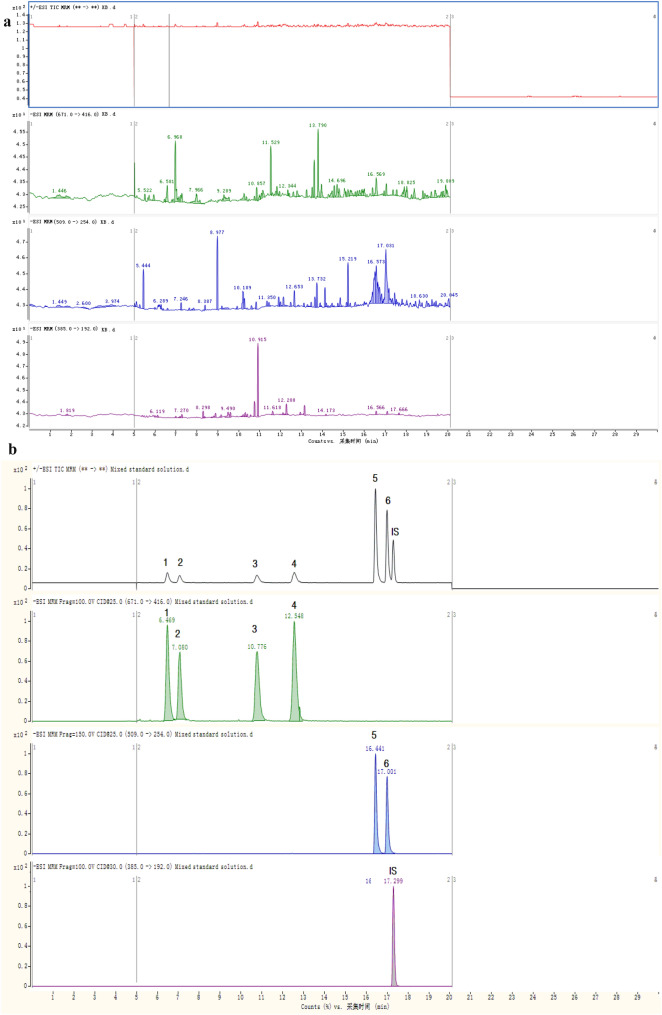

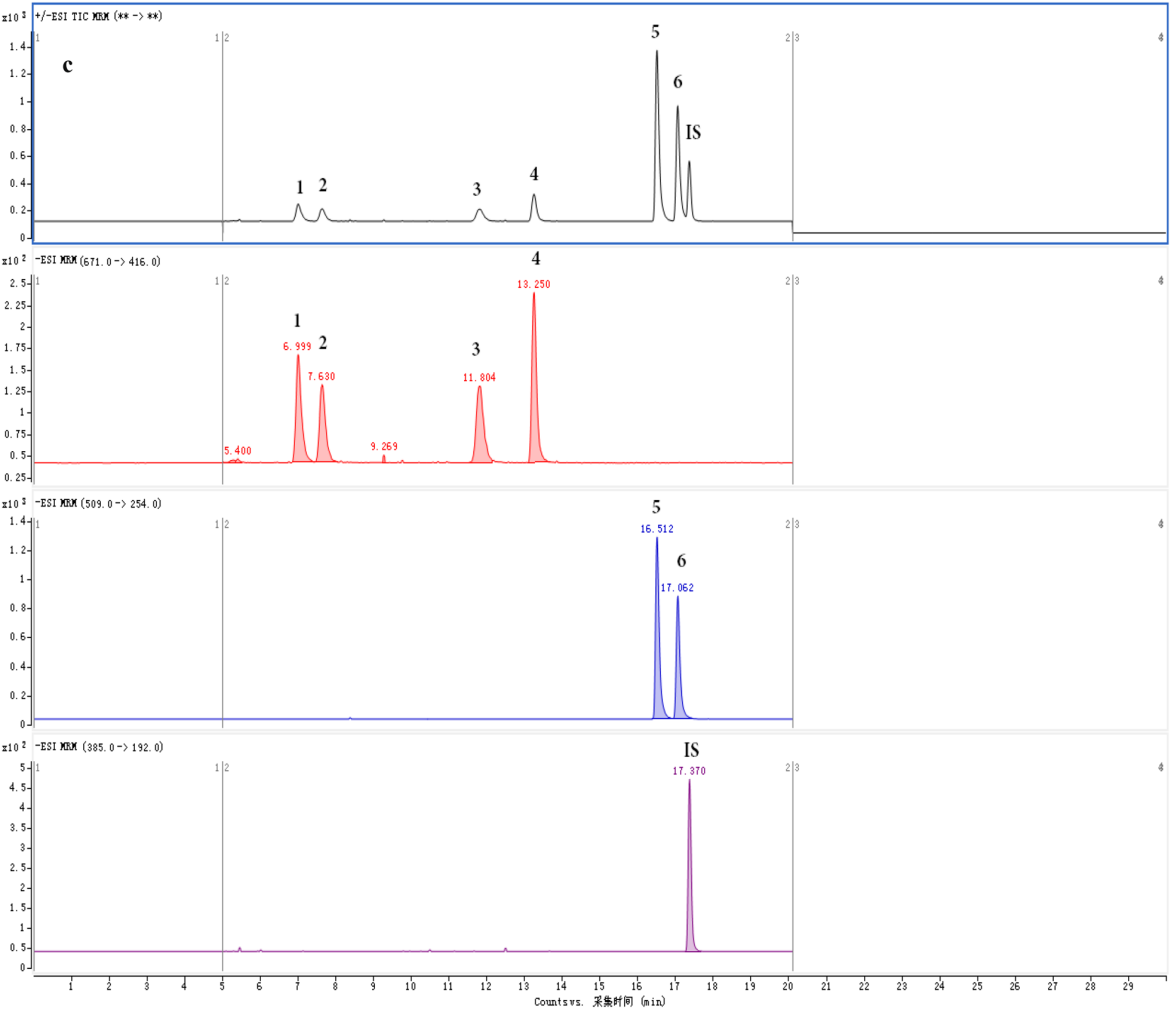
Typical multiple reaction monitoring (MRM) chromatograms for a blank test sample (**A**), a mixed standard solution (**B**) and a sample of *P. multiflorum* (**C**) (1. polygonumnolide C4; 2. polygonumnolide C3; 3. polygonumnolide C1; 4. polygonumnolide C2; 5. *trans*-emodin dianthrones; 6. *cis*-emodin dianthrones; IS. Bianthronyl)

The peaks of the six dianthrones and the IS presented good separation without interference peaks based on the chromatographic and MS conditions mentioned above. The typical MRM chromatograms for a blank test sample, a **mixed standard solution and** a sample of *P. multiflorum* are shown in Fig. 4A–C. This result showed that the method is highly selective.

#### Linearity range, limits of detection (LODs) and limits of quantification (LOQs)

The developed UHPLC-QQQ-MS/MS method was further validated in accordance with the guidelines of the Validation of the Quality Standard of TCMs (Chinese Pharmacopoeia, 2015, volume 1) [3]. Table 3 lists the linear calibration curve with R^2^, linearity range, LOD, and LOQ values. All calibration curves showed good linear regression (r^2^ ≥ 0.9965) within the tested ranges; the LOD (*S/N* = 3) and the LOQ (*S/N* = 10) for the six dianthrones were in the ranges of 0.3–0.4 ng/mL and 0.7–1.1 ng/mL, respectively, showing high sensitivity.

**Table 3:** Regression equation, LOD and LOQ of the six dianthrones

No.	Compounds	Regression equation	R^2^	Range (ng/mL)	LOD (ng/mL)	LOQ (ng/mL)
1	Polygonumnolide C4	Y = 10.186x + 0.0079	0.9980	2.1–126.0	0.4	1.1
2	Polygonumnolide C3	Y = 15.446x + 0.0163	0.9990	2.1–128.4	0.4	1.1
3	Polygonumnolide C1	Y = 17.122x + 0.0534	0.9985	2.8–169.8	0.3	1.1
4	Polygonumnolide C2	Y = 20.117x + 0.0466	0.9983	2.8–168.0	0.3	1.1
5	*trans*-Emodindianthrones	Y = 30.352x + 0.0800	0.9965	3.2–191.0	0.3	0.7
6	*cis*-Emodin dianthrones	Y = 33.308x + 0.0426	0.9978	2.8–168.2	0.3	0.7

#### Precision

The precision of the method was evaluated based on intra- and inter-day precision. The intra-day precision was tested with mixed standard solutions in 1 day. The standard solutions are examined in triplicate on three consecutive days for inter-day precision. The corresponding % RSD values were calculated. The RSDs for the intra-day (n = 6) and inter-day (n = 3) assays were less than 2.73% and 4.63%, respectively (see Table 4).

**Table 4:** Stability, repeatability, precision and recovery of 6 compounds

No	Stability RSD (%) (n = 6)	Repeatability RSD (%) (n = 6)	Precision		Recovery (n = 6)						
			Intra-day RSD (%) (n = 6)	Inter-day RSD (%) (n = 3)	Sample (g)	Original (µg)	Spiked (µg)	Found (µg)	Recovery (%)	Average recovery (%)	RSD (%)
1	3.41	3.12	2.73	2.85	0.502	1.009	1.092	2.383	125.87		
					0.502	1.009	1.092	2.654	150.67		
					0.503	1.011	1.092	2.418	128.81	134.46	7.91
					0.512	1.029	1.092	2.583	142.33		
					0.501	1.007	1.092	2.494	136.14		
					0.518	1.041	1.092	2.383	122.92		
2	3.55	3.24	1.68	2.79	0.502	0.512	0.535	1.184	125.61		
					0.502	0.512	0.535	1.318	150.65		
					0.503	0.513	0.535	1.203	128.97		
					0.512	0.522	0.535	1.328	150.65	134.05	9.70
					0.501	0.511	0.535	1.183	125.61		
					0.518	0.528	0.535	1.185	122.80		
3	3.64	1.92	2.58	2.12	0.502	0.512	0.594	1.345	140.29		
					0.502	0.512	0.594	1.430	154.48		
					0.503	0.513	0.594	1.390	147.60		
					0.512	0.522	0.594	1.454	156.79	150.04	3.88
					0.501	0.511	0.594	1.398	149.35		
					0.518	0.528	0.594	1.430	151.73		
4	3.47	1.23	2.22	4.05	0.502	0.638	0.672	1.505	129.15		
					0.502	0.638	0.672	1.674	154.28		
					0.503	0.639	0.672	1.492	126.94	143.88	8.68
					0.512	0.650	0.672	1.683	153.70		
					0.501	0.636	0.672	1.630	147.93		
					0.518	0.658	0.672	1.674	151.26		
5	3.95	1.97	2.11	4.63	0.502	2.113	2.131	4.159	96.01		
					0.502	2.113	2.131	4.457	109.99		
					0.503	2.118	2.131	4.230	99.11		
					0.512	2.156	2.131	4.430	106.71	105.53	9.65
					0.501	2.109	2.131	4.201	98.17		
					0.518	2.181	2.131	4.806	123.18		
6	3.73	3.30	1.67	2.90	0.502	1.531	1.512	3.051	100.52		
					0.502	1.531	1.512	3.092	103.20		
					0.503	1.534	1.512	3.106	103.98		
					0.512	1.562	1.512	3.289	114.24	104.38	6.19
					0.501	1.528	1.512	2.974	95.66		
					0.518	1.580	1.512	3.224	108.71		

#### Stability and repeatability

The stability was measured using a sample solution and performed at 0, 2, 4, 8, 12, and 24 h after preparation and storage at room temperature. Six independent sample solutions were prepared and analysed to measure the repeatability. The concentration of each solution was determined by calibration curves produced on the same day. The RSDs for stability were less than 3.95% within 24 h. Moreover, the RSDs for repeatability were less than 3.30% (see Table 4). The results of the stability and repeatability tests show that all analytes are stable within the duration of the whole analysis and that the test method is sufficiently effective for conventional analysis.

#### Recovery

The recovery tests were carried out by adding a known number of mixed standards to a certain amount of the six dianthrones. Six replicates were performed for the test. The recoveries were calculated using the following equation: recovery (%) = (total amount detected − amount original)/amount spiked × 100%. Table 4 also shows that the analytical method developed for the six dianthrone compounds has a good recovery rate ranging from 104.38 to 150.04%, and the RSDs were less than 9.70%. Therefore, the UHPLC-QQQ-MS/MS method is precise, accurate, sensitive, and reliable enough for the simultaneous and quantitative determination of the six minor potential hepatotoxic compounds in PMR and PMRP.

### Quantification of the 6 dianthrones in different batches of PMR and PMRP

Comparing the UHPLC retention times and *m/z* values of the six dianthrones with those of the reference compounds, the identification of the target peaks was successful by the UHPLC-QQQ-MS/MS method. The content of each analyte was determined using the respective calibration curves with the IS method. The developed method was successfully applied to analyse the contents of the six dianthrones in PMR and PMRP.

#### Quantification of the 6 dianthrones in 90 batches of PMR

The developed and validated UHPLC-QQQ-MS/MS method was subsequently applied to evaluate the six dianthrones from 90 batches of PMR, and the quantification results are summarized in Table 5. The contents of **1**, **2**, **3**, **4**, **5** and **6** were in the ranges of 0.027–19.04, 0.022–13.86, 0.073–15.53, 0.034–23.35, 0.38–83.67 and 0.29–67.00 µg/g, respectively. The total contents of **1**–**6** ranged from 1.39 to 171.45 µg/g. There were distinct differences in the contents of **1**–**6** in the 90 batches of PMR. Interestingly, the contents of **5** and **6** in PMR extracted with 70% ethanol were remarkably higher than those of **1**–**4**. The average content order in the 90 batches of PMR was **5** > **6** > **1** > **4** > **3** > **2**. According to previous studies [17], dianthrones may be the potential hepatotoxic components in PMR, and **5** and **6** are more toxic than **1**–**4**. Therefore, **5** and **6** could be used as potential toxicity markers of PMRP.

**Table 5:** Contents of 6 dianthrones in 90 batches of Polygoni Multiflori Radix (PMR)

Sample no.	Contents of analytes (µg/g, n = 2^a^)						
	**1**	**2**	**3**	**4**	**5**	**6**	Total
	Mean ± (SD%)^b^	Mean ± (SD%)^b^	Mean ± (SD%)^b^	Mean ± (SD%)^b^	Mean ± (SD%)^b^	Mean ± (SD%)^b^	
PMR-01	2.23 ± 0.09	1.75 ± 0.19	1.81 ± 5.81	1.41 ± 0.73	6.94 ± 0.34	4.96 ± 14.32	19.10
PMR-02	0.84 ± 1.48	0.61 ± 3.34	0.69 ± 0.06	0.81 ± 2.58	1.71 ± 2.59	1.18 ± 1.31	5.84
PMR-03	0.60 ± 1.77	0.47 ± 0.98	0.43 ± 0.93	0.62 ± 3.85	1.22 ± 0.20	0.82 ± 4.54	4.16
PMR-04	19.08 ± 10.75	13.86 ± 11.51	15.53 ± 10.02	12.04 ± 13.86	45.33 ± 41.21	37.33 ± 20.60	143.17
PMR-05	0.83 ± 0.32	0.73 ± 0.63	0.67 ± 2.00	0.78 ± 1.07	6.90 ± 15.20	5.22 ± 16.45	15.13
PMR-06	4.62 ± 0.07	3.99 ± 1.43	4.15 ± 1.22	2.30 ± 0.84	10.37 ± 1.79	9.20 ± 0.35	34.63
PMR-07	0.70 ± 1.03	0.49 ± 0.62	0.56 ± 0.93	0.64 ± 1.93	1.38 ± 0.51	0.68 ± 2.72	4.45
PMR-08	1.43 ± 2.01	0.66 ± 0.36	1.06 ± 0.84	1.30 ± 2.94	4.94 ± 9.23	2.89 ± 4.05	12.28
PMR-09	1.87 ± 0.17	1.21 ± 0.21	1.28 ± 0.23	1.24 ± 2.54	1.55 ± 1.51	1.19 ± 3.36	8.34
PMR-10	3.78 ± 0.35	3.33 ± 0.46	3.57 ± 0.08	1.75 ± 1.60	9.07 ± 1.85	7.08 ± 0.77	28.58
PMR-11	1.52 ± 9.04	1.17 ± 3.03	1.35 ± 1.24	1.45 ± 7.84	8.42 ± 59.40	4.85 ± 5.31	18.76
PMR-12	2.24 ± 5.62	1.51 ± 2.21	1.76 ± 1.00	1.72 ± 2.55	6.51 ± 3.46	5.06 ± 4.21	18.80
PMR-13	2.38 ± 2.36	1.41 ± 0.74	1.64 ± 1.09	2.10 ± 2.36	9.36 ± 81.61	5.75 ± 18.09	22.64
PMR-14	2.56 ± 2.36	1.92 ± 3.47	2.04 ± 1.44	1.55 ± 2.20	3.78 ± 18.58	3.03 ± 10.02	14.88
PMR-15	2.52 ± 0.33	3.03 ± 0.74	2.78 ± 1.67	1.50 ± 0.01	10.64 ± 3.47	10.82 ± 2.61	31.29
PMR-16	7.80 ± 2.01	6.18 ± 2.48	6.49 ± 0.69	4.29 ± 0.92	27.53 ± 4.71	25.28 ± 5.37	77.57
PMR-17	0.14 ± 0.50	0.17 ± 0.34	0.16 ± 0.35	0.037 ± 0.16	1.01 ± 10.91	0.46 ± 1.36	1.98
PMR-18	0.25 ± 0.60	0.022 ± 0.00	0.073 ± 0.00	0.74 ± 0.03	1.01 ± 6.87	0.82 ± 1.96	2.92
PMR-19	0.69 ± 0.14	0.52 ± 0.16	0.33 ± 0.63	1.62 ± 2.82	7.31 ± 40.20	5.35 ± 14.37	15.82
PMR-20	5.91 ± 0.12	0.94 ± 0.54	0.69 ± 0.04	3.90 ± 0.17	64.18 ± 8.23	48.48 ± 3.40	124.1
PMR-21	0.20 ± 0.88	2.82 ± 0.33	5.10 ± 5.70	0.50 ± 0.74	4.15 ± 5.15	2.84 ± 0.01	15.61
PMR-22	0.027 ± 0.28	0.48 ± 0.75	0.27 ± 0.92	0.034 ± 0.72	0.47 ± 2.22	0.29 ± 2.25	1.57
PMR-23	0.042 ± 0.10	0.11 ± 0.71	0.10 ± 0.08	0.10 ± 0.43	0.63 ± 3.82	0.41 ± 0.62	1.39
PMR-24	1.87 ± 2.82	0.181 ± 0.33	0.13 ± 0.30	2.04 ± 0.61	2.54 ± 3.68	1.84 ± 0.45	8.60
PMR-25	2.58 ± 0.30	1.091 ± 0.00	1.42 ± 3.62	23.35 ± 19.62	18.06 ± 1.04	13.68 ± 2.07	60.18
PMR-26	0.55 ± 0.02	2.211 ± 0.02	2.63 ± 0.33	1.51 ± 0.12	22.66 ± 7.04	20.44 ± 0.09	50.00
PMR-27	0.42 ± 1.45	0.791 ± 0.10	1.18 ± 0.19	1.54 ± 2.72	8.24 ± 22.57	5.47 ± 18.23	17.64
PMR-28	0.48 ± 0.29	1.22 ± 4.70	0.57 ± 0.19	1.20 ± 0.37	7.93 ± 10.35	6.24 ± 16.38	17.64
PMR-29	0.054 ± 0.03	0.76 ± 1.43	0.54 ± 0.79	0.037 ± 0.39	4.17 ± 2.97	3.31 ± 2.33	8.87
PMR-30	0.27 ± 0.54	0.13 ± 0.53	0.11 ± 0.18	0.15 ± 0.42	1.03 ± 0.08	0.82 ± 1.30	2.51
PMR-31	5.20 ± 1.85	0.19 ± 0.06	0.27 ± 0.02	3.96 ± 0.21	10.74 ± 2.01	8.84 ± 3.84	29.20
PMR-32	1.27 ± 0.35	2.89 ± 3.50	3.22 ± 7.05	0.92 ± 2.08	5.12 ± 5.83	4.66 ± 6.80	18.08
PMR-33	6.83 ± 0.15	0.83 ± 0.97	0.95 ± 1.35	5.82 ± 2.78	51.11 ± 13.81	42.50 ± 3.46	108.04
PMR-34	4.89 ± 1.01	6.02 ± 0.36	6.52 ± 0.38	3.41 ± 2.06	21.71 ± 2.04	15.11 ± 4.99	57.66
PMR-35	7.67 ± 0.42	3.28 ± 0.48	4.34 ± 0.09	5.81 ± 0.60	61.94 ± 0.67	50.02 ± 5.90	133.06
PMR-36	3.38 ± 1.19	6.11 ± 2.08	7.37 ± 0.36	1.73 ± 0.82	16.51 ± 0.62	12.09 ± 0.64	47.19
PMR-37	0.54 ± 0.63	2.47 ± 0.08	3.28 ± 0.33	0.32 ± 2.23	2.12 ± 6.34	1.55 ± 2.14	10.28
PMR-38	4.17 ± 10.69	0.36 ± 1.36	0.48 ± 0.03	2.50 ± 1.15	8.77 ± 5.04	7.51 ± 0.86	23.79
PMR-39	2.01 ± 0.33	2.81 ± 8.05	3.23 ± 4.53	1.13 ± 3.14	5.79 ± 38.26	5.58 ± 11.65	20.55
PMR-40	1.56 ± 0.23	1.37 ± 1.06	1.56 ± 4.16	1.03 ± 0.20	7.07 ± 20.29	5.61 ± 9.30	18.20
PMR-41	6.02 ± 11.72	1.113 ± 0.38	1.32 ± 5.52	3.75 ± 1.79	7.71 ± 6.91	6.26 ± 21.43	26.17
PMR-42	4.68 ± 3.66	3.85 ± 5.45	4.47 ± 2.27	2.86 ± 1.09	6.45 ± 1.67	5.70 ± 23.76	28.01
PMR-43	10.39 ± 0.67	3.16 ± 2.64	3.58 ± 14.24	9.88 ± 0.86	27.34 ± 7.43	20.75 ± 0.12	75.10
PMR-44	1.25 ± 3.60	0.79 ± 3.24	0.66 ± 0.91	1.34 ± 2.87	2.51 ± 2.49	1.87 ± 4.10	8.42
PMR-45	0.92 ± 0.18	0.60 ± 1.06	0.59 ± 0.88	0.64 ± 1.18	4.68 ± 1.77	3.27 ± 17.57	10.7
PMR-46	1.28 ± 0.74	0.83 ± 1.20	0.74 ± 1.03	1.12 ± 0.98	2.35 ± 4.83	1.68 ± 3.90	8.00
PMR-47	2.26 ± 1.93	1.34 ± 3.33	1.35 ± 0.94	1.71 ± 1.61	9.27 ± 16.24	6.60 ± 6.12	22.53
PMR-48	1.45 ± 2.29	0.88 ± 1.09	0.91 ± 0.03	0.72 ± 3.24	7.18 ± 22.72	6.43 ± 31.68	17.57
PMR-49	1.70 ± 0.85	0.93 ± 0.33	1.02 ± 0.67	1.14 ± 2.18	6.03 ± 0.71	4.94 ± 13.15	15.76
PMR-50	0.94 ± 0.95	0.53 ± 1.70	0.55 ± 0.99	0.64 ± 0.69	4.48 ± 21.93	3.16 ± 15.33	10.3
PMR-51	1.22 ± 1.04	0.65 ± 1.13	0.76 ± 1.04	0.80 ± 2.97	6.91 ± 26.37	6.53 ± 28.27	16.87
PMR-52	1.30 ± 4.47	0.80 ± 1.93	0.74 ± 3.43	0.74 ± 4.23	3.35 ± 18.15	2.60 ± 17.49	9.53
PMR-53	7.62 ± 0.08	0.69 ± 0.19	5.63 ± 0.90	7.22 ± 0.34	47.24 ± 6.47	38.91 ± 5.55	107.31
PMR-54	3.01 ± 0.17	3.30 ± 0.17	2.42 ± 1.11	2.85 ± 0.93	38.15 ± 9.29	33.72 ± 1.68	83.45
PMR-55	6.76 ± 7.02	4.92 ± 7.27	3.93 ± 4.57	5.17 ± 5.17	83.67 ± 3.75	67.00 ± 9.16	171.45
PMR-56	0.32 ± 0.51	0.22 ± 1.18	0.17 ± 0.14	0.28 ± 1.06	6.78 ± 28.19	4.95 ± 6.74	12.72
PMR-57	0.61 ± 0.50	0.35 ± 0.20	0.37 ± 0.81	0.42 ± 0.51	1.59 ± 0.79	1.35 ± 0.16	4.69
PMR-58	1.25 ± 1.12	0.92 ± 0.54	0.81 ± 2.83	0.95 ± 0.48	9.33 ± 11.15	6.29 ± 37.35	19.55
PMR-59	1.29 ± 0.21	0.90 ± 3.74	0.85 ± 0.82	0.91 ± 1.22	9.48 ± 9.83	6.30 ± 15.03	19.73
PMR-60	1.46 ± 0.75	1.09 ± 2.17	0.96 ± 1.72	1.03 ± 3.02	9.51 ± 5.22	6.47 ± 4.66	20.52
PMR-61	3.99 ± 1.92	2.63 ± 2.76	2.30 ± 4.61	2.31 ± 0.92	12.10 ± 15.76	9.72 ± 19.35	33.05
PMR-62	1.77 ± 4.12	1.01 ± 0.31	1.03 ± 2.95	1.15 ± 1.08	4.55 ± 12.20	3.83 ± 8.55	13.34
PMR-63	0.82 ± 1.08	0.81 ± 0.66	0.54 ± 1.22	0.80 ± 0.91	5.39 ± 1.52	4.24 ± 5.97	12.6
PMR-64	0.46 ± 0.91	0.29 ± 0.98	0.27 ± 0.13	0.31 ± 0.08	1.42 ± 1.13	1.40 ± 0.53	4.15
PMR-65	1.41 ± 0.36	0.94 ± 0.51	1.02 ± 0.07	1.37 ± 0.21	7.01 ± 0.84	6.76 ± 3.08	18.51
PMR-66	1.16 ± 1.65	0.72 ± 0.30	0.71 ± 0.35	0.74 ± 2.46	3.84 ± 5.18	3.34 ± 9.40	10.51
PMR-67	1.47 ± 2.11	0.99 ± 0.30	0.91 ± 2.47	0.82 ± 0.98	7.48 ± 22.19	6.91 ± 12.61	18.58
PMR-68	1.60 ± 0.24	0.97 ± 1.97	0.92 ± 6.10	1.01 ± 2.62	2.57 ± 11.58	2.03 ± 13.81	9.10
PMR-69	0.77 ± 0.25	0.51 ± 0.16	0.52 ± 0.14	0.78 ± 0.34	8.49 ± 2.12	8.84 ± 0.66	19.91
PMR-70	0.38 ± 0.35	0.16 ± 0.51	0.18 ± 0.64	0.39 ± 0.26	3.46 ± 9.85	2.66 ± 2.92	7.23
PMR-71	1.11 ± 5.67	0.71 ± 4.43	0.67 ± 1.40	0.74 ± 5.16	3.50 ± 19.82	2.38 ± 21.85	9.11
PMR-72	0.96 ± 2.83	0.80 ± 2.48	0.68 ± 0.03	0.72 ± 3.11	7.88 ± 15.41	5.45 ± 6.51	16.49
PMR-73	0.75 ± 0.27	0.41 ± 0.64	0.37 ± 0.99	0.79 ± 0.33	3.37 ± 7.62	2.49 ± 7.62	8.18
PMR-74	2.63 ± 0.78	0.97 ± 1.51	1.46 ± 0.24	2.98 ± 0.64	19.43 ± 1.13	14.40 ± 2.76	41.87
PMR-75	2.34 ± 0.29	1.42 ± 2.21	0.91 ± 1.94	2.22 ± 0.21	66.05 ± 13.61	41.98 ± 3.82	114.92
PMR-76	0.25 ± 0.24	0.12 ± 1.35	0.15 ± 0.11	0.23 ± 0.18	1.26 ± 2.63	0.83 ± 0.57	2.84
PMR-77	0.46 ± 0.35	0.39 ± 1.12	0.25 ± 0.36	0.40 ± 1.89	3.92 ± 0.17	3.14 ± 2.26	8.56
PMR-78	1.14 ± 1.65	0.74 ± 2.18	0.66 ± 0.99	0.70 ± 1.99	3.03 ± 15.82	2.38 ± 17.31	8.65
PMR-79	0.61 ± 4.10	0.49 ± 4.96	0.34 ± 4.95	0.40 ± 0.26	5.46 ± 11.44	3.59 ± 9.55	10.89
PMR-80	0.26 ± 0.66	0.15 ± 0.62	0.12 ± 0.07	0.27 ± 0.10	0.38 ± 0.31	0.37 ± 0.88	1.55
PMR-81	0.67 ± 0.17	0.35 ± 0.60	0.45 ± 0.13	0.92 ± 0.24	8.68 ± 3.51	7.25 ± 3.26	18.32
PMR-82	4.59 ± 0.34	3.78 ± 1.03	3.21 ± 1.38	4.14 ± 0.94	42.84 ± 8.21	37.40 ± 1.94	95.96
PMR-83	0.18 ± 2.23	0.089 ± 1.91	0.10 ± 1.67	0.14 ± 0.07	1.47 ± 1.69	0.94 ± 0.42	2.919
PMR-84	2.28 ± 0.34	1.15 ± 1.18	1.35 ± 0.89	1.95 ± 0.26	11.61 ± 11.10	9.29 ± 7.78	27.63
PMR-85	0.84 ± 0.09	0.47 ± 0.14	0.51 ± 0.87	0.82 ± 0.39	12.74 ± 2.17	12.40 ± 1.07	27.78
PMR-86	2.06 ± 1.19	1.31 ± 3.95	1.22 ± 3.72	1.33 ± 5.86	3.97 ± 21.77	3.47 ± 18.51	13.36
PMR-87	6.11 ± 0.10	5.20 ± 1.19	4.61 ± 0.93	4.82 ± 0.22	52.09 ± 15.47	46.51 ± 7.87	119.34
PMR-88	0.89 ± 6.61	0.53 ± 2.83	0.50 ± 5.50	0.52 ± 1.83	3.23 ± 5.44	1.99 ± 0.73	7.66
PMR-89	2.13 ± 1.71	1.35 ± 0.48	1.22 ± 5.08	1.28 ± 1.00	4.92 ± 1.91	3.69 ± 4.18	14.59
PMR-90	0.98 ± 0.36	0.65 ± 2.25	0.59 ± 1.27	0.60 ± 1.91	4.44 ± 17.23	3.93 ± 19.42	11.19
Average	2.30	1.53	1.67	1.99	12.23	9.75	29.46

^a^The data are presented as the average of two replicates

^b^SD% is presented in the table

#### Quantification of the 6 dianthrones in different batches of PMRP (the water-steaming method)

The developed and validated UHPLC-QQQ-MS/MS method was subsequently applied to identify the six dianthrones in 10 samples of PMRP (PMRP-S_0h_, PMRP-S_2h_, PMRP-S_4h_, PMRP-S_6h_, PMRP-S_8h_, PMRP-S_10h_, PMRP-S_12h_, PMRP-S_16h_, PMRP-S_20h_, and PMRP-S_24h_) using the water-steaming method, and the quantification results are summarized in Table 6. The contents of **1**, **2**, **3**, **4**, **5** and **6** were in the ranges of 0.18–2.09, 0.58–3.67, 0.26–2.04, 0.71–4.07, 0.25–7.20 and 0.22–6.11 µg/g, respectively. The total contents of **1–6** ranged from 2.20 to 20.74 µg/g.

**Table 6:** Contents of 6 dianthrones in 10 samples of Polygoni Multiflori Radix Praeparata (PMRP) with the water steaming method

Sample no.	Contents of analytes (ug/g, n = 2^a^)						
	**1**	**2**	**3**	**4**	**5**	**6**	Total
	Mean ± (SD%)^b^	Mean ± (SD%)^b^	Mean ± (SD%)^b^	Mean ± (SD%)^b^	Mean ± (SD%)^b^	Mean ± (SD%)^b^	
PMRP -S_0h_	2.09 ± 1.53	1.65 ± 2.19	2.04 ± 031	1.65 ± 4.09	7.20 ± 11.48	6.11 ± 14.10	20.74
PMRP -S_2h_	1.46 ± 1.16	1.99 ± 4.56	1.68 ± 2.18	1.87 ± 1.66	0.94 ± 1.97	0.83 ± 1.70	8.77
PMRP -S_4h_	1.13 ± 0.32	2.79 ± 0.38	1.66 ± 2.35	3.09 ± 3.75	0.81 ± 1.44	0.71 ± 2.17	10.19
PMRP -S_6h_	1.26 ± 2.38	3.67 ± 4.92	1.48 ± 0.67	4.07 ± 0.28	0.88 ± 2.86	0.84 ± 1.44	12.20
PMRP -S_8h_	0.61 ± 1.18	1.24 ± 0.24	0.87 ± 0.73	1.67 ± 0.21	0.28 ± 0.78	0.25 ± 1.41	4.92
PMRP -S_10h_	0.85 ± 1.90	1.49 ± 0.58	1.13 ± 2.05	1.7 ± 3.05	0.41 ± 0.22	0.36 ± 0.66	5.94
PMRP-S_12h_	0.66 ± 0.65	1.27 ± 1.36	0.91 ± 1.55	1.65 ± 1.57	0.36 ± 0.78	0.31 ± 0.77	5.16
PMRP-S_16h_	0.52 ± 1.98	1.37 ± 2.93	0.61 ± 1.96	1.52 ± 1.79	0.39 ± 0.90	0.35 ± 1.57	4.76
PMRP-S_20h_	0.39 ± 0.23	0.92 ± 0.51	0.49 ± 0.075	1.29 ± 0.20	0.47 ± 0.63	0.42 ± 0.37	3.98
PMRP-S_24h_	0.18 ± 0.15	0.58 ± 1.20	0.26 ± 0.22	0.71 ± 0.72	0.25 ± 0.16	0.22 ± 0.37	2.20

^a^The data are presented as the average of two replicates

^b^SD% is presented in the table

Additionally, the developed and validated UHPLC-QQQ-MS/MS method was subsequently applied to determine the contents of the six dianthrones in 45 samples of PMRP after 0, 12, and 24 h of processing using the water-steaming method, and the quantification results are summarized in Table 7. The total contents of **1**–**6** were found to have decreased significantly. Compounds **1** and** 3** could be detected in 5 samples after 12 h of processing, Compound **1** could not be detected in 15 samples, and compound **2** could not be detected in 6 samples after 12 h of processing. However, compounds **5** and **6** could be detected in all samples after 12 h of processing, with the contents of **5** ranging from 0.17 to 0.78 µg/g and those of **6** ranging from 0.14 to 0.73 µg/g Finally, after 24 h of processing, the contents of the six dianthrones all decreased by more than 80%.

**Table 7:** Contents of 6 compounds in 45 samples of Polygoni Multiflori Radix Praeparata (PMRP) with the water steaming method

Sample no.	Contents of analytes (µg/g, n = 2^a^)																	
	**1**			**2**			**3**			**4**			**5**			**6**		
	0 h	12 h	24 h	0 h	12 h	24 h	0 h	12 h	24 h	0 h	12 h	24 h	0 h	12 h	24 h	0 h	12 h	24 h
PMRP-SZ01	0.37	ND^b^	ND^b^	0.32	0.11	ND^b^	0.37	ND^b^	ND^b^	0.32	0.097	ND^b^	1.05	0.49	0.22	0.68	0.40	0.18
PMRP-SZ02	0.157	ND^b^	ND^b^	0.19	0.12	ND^b^	0.19	ND^b^	ND^b^	0.19	0.10	ND^b^	1.34	0.45	0.17	0.85	0.38	0.14
PMRP-SZ03	1.42	0.36	ND^b^	1.06	0.16	ND^b^	1.18	0.099	ND^b^	0.94	0.16	ND^b^	6.39	0.32	0.20	6.26	0.27	0.16
PMRP-SZ04	0.58	ND^b^	ND^b^	0.45	0.11	ND^b^	0.52	ND^b^	ND^b^	0.50	0.10	ND^b^	1.45	0.29	0.23	1.22	0.25	0.18
PMRP-SZ05	0.72	0.020	ND^b^	0.50	0.16	0.11	0.51	0.089	ND^b^	0.90	0.14	0.085	2.95	0.62	0.29	2.26	0.44	0.24
PMRP-SZ06	0.023	ND^b^	ND^b^	0.13	0.10	0.089	0.089	ND^b^	ND^b^	0.10	0.088	0.070	0.68	0.21	0.16	0.54	0.17	0.12
PMRP-SZ07	0.43	0.04	ND^b^	0.47	1.05	0.12	0.46	0.22	ND^b^	0.49	0.19	0.093	3.73	0.72	0.26	3.41	0.62	0.21
PMRP-SZ08	3.89	0.088	ND^b^	2.67	0.24	0.12	2.91	0.16	ND^b^	2.60	0.25	0.097	10.33	1.69	0.24	8.87	1.53	0.18
PMRP-SZ09	0.92	0.035	ND^b^	0.88	0.15	0.11	0.90	0.11	ND^b^	0.83	0.18	0.088	6.74	0.69	0.21	4.95	0.54	0.17
PMRP-SZ10	1.21	0.060	ND^b^	1.96	0.22	0.11	1.85	0.13	ND^b^	1.87	0.30	0.081	6.99	1.74	0.78	6.11	1.25	0.73
PMRP-SZ11	1.24	0.052	ND^b^	0.98	0.21	0.10	1.12	0.13	ND^b^	1.04	0.25	0.082	8.11	1.19	0.26	5.74	1.01	0.20
PMRP-SZ12	1.99	0.31	ND^b^	1.38	0.43	0.10	1.57	0.31	ND^b^	1.51	0.42	ND^b^	3.46	3.04	0.24	3.16	2.77	0.18
PMRP-SZ13	1.21	0.021	ND^b^	1.00	0.14	ND^b^	1.07	0.099	ND^b^	1.09	0.14	ND^b^	8.01	2.99	0.55	5.75	1.92	0.46
PMRP-SZ14	1.41	0.022	ND^b^	1.17	0.13	ND^b^	1.25	0.10	ND^b^	1.17	0.14	ND^b^	8.12	0.54	0.39	5.88	0.45	0.29
PMRP-SZ15	2.41	ND^b^	ND^b^	1.99	0.14	0.10	2.39	ND^b^	ND^b^	3.67	0.12	0.085	17.52	6.87	0.34	13.11	4.76	0.27

^a^The data are presented as the average of two replicates

^b^ND, under limits of quantitation

#### Quantification of the 6 dianthrones in 86 batches of commercial PMRP

The developed and validated UHPLC-QQQ-MS/MS method was subsequently applied to determine the contents of the six dianthrones in 86 batches of commercial PMRP, and the quantification results are summarized in Table 8. The contents of **1**, **2**, **3**, **4**, **5** and **6** were in the ranges of 0.020–13.03, 0.051–8.94, 0.022–7.23, 0.030–12.75, 0.098–28.54 and 0.14–27.79 µg/g, respectively. The total contents of **1**–**6** ranged from 0.35 to 65.27 µg/g. There were distinct differences in the contents of compounds **1**–**6** in the 86 batches of commercial PMRP. Interestingly, the contents of **5** and **6** in the PMR sample extracted with 70% ethanol were remarkably higher than those of **1**–**4**. The average content order in the 86 batches of PMR was **5** > **6** > **4** > **2** > **1** > **3**.

**Table 8:** Contents of 6 dianthrones in 86 batches of Polygoni Multiflori Radix Praeparata (PMRP)

Sample no.	Contents of analytes (µg/g, n = 2^a^)						
	**1**	**2**	**3**	**4**	**5**	**6**	Total
	Mean ± (SD%)^c^	Mean ± (SD%)^c^	Mean ± (SD%)^c^	Mean ± (SD%)^c^	Mean ± (SD%)^c^	Mean ± (SD%)^c^	
PMRP-01	0.60 ± 1.32	3.36 ± 1.60	0.76 ± 1.04	2.57 ± 5.40	2.41 ± 6.96	2.16 ± 5.87	11.86
PMRP-02	0.051 ± 0.56	0.070 ± 0.98	0.064 ± 0.61	0.069 ± 0.73	0.41 ± 1.10	0.36 ± 0.74	1.02
PMRP-03	0.25 ± 0.42	0.45 ± 1.68	0.26 ± 1.15	0.60 ± 0.79	4.50 ± 12.09	3.38 ± 5.03	9.44
PMRP-04	0.051 ± 0.04	0.078 ± 0.15	0.071 ± 0.26	0.11 ± 0.59	0.35 ± 0.66	0.36 ± 0.78	1.02
PMRP-05	0.17 ± 0.23	0.23 ± 0.33	0.18 ± 0.11	0.35 ± 0.29	7.05 ± 17.31	6.70 ± 6.99	14.68
PMRP-06	0.081 ± 0.25	0.071 ± 0.09	0.084 ± 0.26	0.082 ± 0.17	5.47 ± 0.75	4.60 ± 17.77	10.39
PMRP-07	0.12 ± 0.14	0.19 ± 0.11	0.13 ± 0.11	0.28 ± 0.04	0.36 ± 0.83	0.37 ± 1.62	1.45
PMRP-08	0.16 ± 0.49	0.36 ± 0.70	0.19 ± 0.11	0.47 ± 0.06	7.21 ± 8.07	5.10 ± 27.48	13.49
PMRP-09	0.16 ± 0.09	0.47 ± 1.30	0.18 ± 0.57	0.42 ± 0.48	6.30 ± 9.12	6.09 ± 18.05	13.62
PMRP-10	0.079 ± 0.09	0.13 ± 0.24	0.094 ± 0.02	0.17 ± 0.99	0.19 ± 0.17	0.24 ± 0.61	0.90
PMRP-11	7.46 ± 2.35	5.73 ± 3.87	4.23 ± 0.28	7.36 ± 4.25	9.47 ± 4.41	8.05 ± 38.33	42.30
PMRP-12	0.10 ± 0.06	0.34 ± 0.26	0.11 ± 0.07	0.27 ± 0.19	1.25 ± 0.88	1.23 ± 2.59	3.30
PMRP-13	0.030 ± 0.00	^b^ND	0.05 ± 0.00	0.031 ± 0.00	0.098 ± 0.12	0.14 ± 0.58	0.35
PMRP-14	0.090 ± 0.22	0.13 ± 0.65	0.11 ± 0.40	0.16 ± 0.30	2.54 ± 1.95	2.27 ± 2.78	5.30
PMRP-15	3.51 ± 4.24	3.08 ± 2.97	2.47 ± 3.51	4.37 ± 9.27	2.68 ± 5.17	2.12 ± 4.77	18.23
PMRP-16	0.062 ± 0.05	0.095 ± 0.15	0.072 ± 0.34	0.085 ± 0.17	4.08 ± 5.99	3.99 ± 3.69	8.38
PMRP-17	0.070 ± 0.35	0.088 ± 0.23	0.081 ± 0.02	0.10 ± 0.34	0.62 ± 2.46	0.62 ± 1.38	1.58
PMRP-18	0.047 ± 0.26	0.082 ± 0.37	0.067 ± 0.26	0.098 ± 0.22	0.24 ± 0.20	0.27 ± 0.31	0.80
PMRP-19	4.42 ± 2.17	4.67 ± 4.59	2.36 ± 5.45	6.42 ± 4.79	8.68 ± 7.05	8.38 ± 28.49	34.93
PMRP-20	0.51 ± 0.27	0.44 ± 0.35	0.67 ± 0.15	0.64 ± 0.10	28.54 ± 7.99	25.95 ± 5.23	56.75
PMRP-21	0.047 ± 0.26	0.051 ± 0.19	0.074 ± 0.55	0.063 ± 0.65	0.52 ± 0.61	0.45 ± 0.39	1.21
PMRP-22	0.22 ± 0.12	0.29 ± 0.00	0.20 ± 0.00	0.40 ± 0.32	9.31 ± 0.00	8.62 ± 0.00	19.04
PMRP-23	0.030 ± 0.08	^b^ND	0.050 ± 0.00	0.030 ± 0.45	0.19 ± 2.99	0.21 ± 2.62	0.51
PMRP-24	0.77 ± 0.99	0.64 ± 0.49	0.46 ± 1.93	0.99 ± 1.74	8.87 ± 10.19	8.22 ± 2.72	19.95
PMRP-25	0.14 ± 0.17	0.28 ± 0.44	0.18 ± 0.62	0.34 ± 0.92	2.81 ± 12.24	2.02 ± 4.31	5.77
PMRP-26	0.10 ± 0.06	0.068 ± 0.04	0.095 ± 0.37	0.098 ± 0.11	0.71 ± 0.52	0.63 ± 1.47	1.70
PMRP-27	0.26 ± 0.59	0.42 ± 0.96	0.27 ± 0.02	0.67 ± 1.75	6.52 ± 5.11	5.13 ± 19.97	13.27
PMRP-28	1.18 ± 0.03	1.75 ± 0.53	1.35 ± 0.05	2.35 ± 0.45	13.77 ± 7.12	10.30 ± 6.31	30.7
PMRP-29	0.063 ± 0.11	0.14 ± 0.39	0.091 ± 0.07	0.16 ± 0.52	0.95 ± 5.51	0.71 ± 2.44	2.11
PMRP-30	0.054 ± 0.29	0.16 ± 0.60	0.098 ± 1.79	0.12 ± 0.08	1.53 ± 3.12	1.19 ± 3.69	3.15
PMRP-31	9.37 ± 8.79	6.32 ± 11.82	5.23 ± 7.88	11.36 ± 14.95	8.23 ± 1.64	7.79 ± 6.42	48.30
PMRP-32	3.48 ± 1.90	2.02 ± 0.60	1.70 ± 1.63	3.40 ± 1.31	6.73 ± 3.92	5.92 ± 22.20	23.25
PMRP-33	0.065 ± 0.41	0.094 ± 0.21	0.084 ± 0.28	0.12 ± 0.19	0.59 ± 5.10	0.51 ± 3.46	1.46
PMRP-34	0.081 ± 0.57	0.10 ± 0.58	0.085 ± 0.27	0.12 ± 0.59	0.89 ± 3.39	0.70 ± 3.10	1.98
PMRP-35	0.16 ± 0.81	0.23 ± 0.18	0.17 ± 0.30	0.41 ± 1.48	0.32 ± 1.99	0.35 ± 1.18	1.64
PMRP-36	0.15 ± 0.25	0.27 ± 0.02	0.17 ± 0.07	0.34 ± 0.03	0.31 ± 0.03	3.52 ± 9.47	4.76
PMRP-37	0.11 ± 0.02	0.34 ± 0.14	0.12 ± 0.09	0.35 ± 0.49	0.33 ± 1.51	0.31 ± 1.16	1.56
PMRP-38	0.056 ± 0.01	0.075 ± 0.29	0.071 ± 0.21	0.089 ± 0.01	0.28 ± 0.15	0.30 ± 1.38	0.87
PMRP-39	0.21 ± 0.64	0.53 ± 0.51	0.23 ± 0.15	0.59 ± 1.60	3.95 ± 12.81	2.67 ± 2.57	8.18
PMRP-40	0.057 ± 0.08	0.18 ± 0.83	0.072 ± 0.15	0.12 ± 0.36	3.24 ± 9.23	2.66 ± 3.43	6.33
PMRP-41	0.025 ± 0.29	0.051 ± 0.19	0.039 ± 0.13	0.063 ± 0.31	0.38 ± 0.63	0.27 ± 0.86	0.83
PMRP-42	^b^ND	0.089 ± 0.00	^b^ND	0.14 ± 0.02	0.36 ± 2.36	0.24 ± 0.90	0.83
PMRP-43	0.13 ± 0.028	0.39 ± 0.32	0.16 ± 1.60	0.62 ± 0.53	1.42 ± 10.91	1.13 ± 14.98	3.85
PMRP-44	0.025 ± 0.08	0.17 ± 0.061	0.054 ± 0.13	0.23 ± 0.11	0.32 ± 0.70	0.21 ± 0.15	1.01
PMRP-45	^b^ND	0.089 ± 0.043	^b^ND	0.14 ± 0.00	0.33 ± 0.26	0.24 ± 0.71	0.80
PMRP-46	0.028 ± 0.17	0.48 ± 0.028	0.14 ± 0.37	0.56 ± 0.90	3.25 ± 16.03	3.29 ± 7.35	7.86
PMRP-47	0.08 ± 0.16	0.411 ± 0.08	0.073 ± 0.44	0.32 ± 0.14	0.40 ± 1.05	0.28 ± 0.35	1.53
PMRP-48	0.42 ± 0.04	1.63 ± 1.42	0.69 ± 0.28	2.68 ± 0.31	9.86 ± 0.14	7.38 ± 1.26	22.66
PMRP-49	0.20 ± 0.03	1.21 ± 0.04	0.36 ± 0.00	1.59 ± 0.02	26.99 ± 10.44	27.79 ± 0.27	58.14
PMRP-50	7.40 ± 2.13	5.61 ± 0.37	3.94 ± 0.39	10.31 ± 1.79	11.01 ± 0.97	10.85 ± 0.43	49.12
PMRP-51	0.027 ± 0.38	0.13 ± 0.01	0.040 ± 0.07	0.17 ± 0.18	1.90 ± 1.42	1.53 ± 0.53	3.80
PMRP-52	13.03 ± 18.67	8.94 ± 20.21	7.23 ± 10.11	12.75 ± 16.48	10.82 ± 61.54	12.50 ± 6.99	65.27
PMRP-53	0.46 ± 0.41	1.06 ± 3.01	0.54 ± 0.34	1.35 ± 3.72	1.26 ± 3.35	1.41 ± 2.37	6.08
PMRP-54	0.16 ± 0.08	0.54 ± 1.02	0.20 ± 0.36	0.64 ± 0.52	5.29 ± 7.85	5.50 ± 14.07	12.33
PMRP-55	0.072 ± 0.08	0.36 ± 0.60	0.10 ± 0.03	0.41 ± 0.64	1.81 ± 4.11	1.80 ± 1.06	4.55
PMRP-56	8.25 ± 4.61	4.64 ± 5.20	4.95 ± 5.92	8.79 ± 10.87	3.80 ± 7.98	4.60 ± 2.97	35.03
PMRP-57	0.22 ± 0.78	0.58 ± 1.31	0.25 ± 0.63	0.79 ± 1.61	5.18 ± 12.17	4.65 ± 3.99	11.67
PMRP-58	0.40 ± 0.89	0.92 ± 4.89	0.55 ± 0.53	1.15 ± 0.59	3.24 ± 0.48	3.41 ± 6.09	9.67
PMRP-59	0.71 ± 0.64	0.87 ± 5.14	0.54 ± 0.26	0.92 ± 1.15	16.79 ± 24.06	18.91 ± 70.89	38.74
PMRP-60	5.61 ± 0.64	8.19 ± 2.47	3.68 ± 0.55	10.28 ± 0.99	18.71 ± 9.53	18.75 ± 7.29	65.22
PMRP-61	0.032 ± 0.18	0.17 ± 0.24	0.054 ± 0.23	0.21 ± 0.66	0.29 ± 2.00	0.18 ± 1.71	0.94
PMRP-62	0.095 ± 0.10	0.16 ± 0.75	0.069 ± 0.43	0.25 ± 0.27	0.34 ± 3.00	0.24 ± 1.83	1.15
PMRP-63	0.13 ± 0.07	0.57 ± 0.69	0.13 ± 0.09	0.49 ± 0.13	0.56 ± 0.41	0.46 ± 1.84	2.34
PMRP-64	^b^ND	0.12 ± 0.43	0.032 ± 0.10	0.17 ± 0.48	0.26 ± 0.62	0.15 ± 0.60	0.73
PMRP-65	0.028 ± 0.17	0.11 ± 0.12	0.044 ± 0.04	0.17 ± 0.27	0.49 ± 0.31	0.40 ± 1.86	1.24
PMRP-66	0.08 ± 0.36	0.15 ± 0.10	0.040 ± 0.24	0.20 ± 1.23	0.28 ± 0.09	0.19 ± 0.92	0.89
PMRP-67	0.52 ± 1.64	0.36 ± 0.38	0.31 ± 0.04	0.73 ± 1.15	0.38 ± 0.14	0.31 ± 0.58	2.61
PMRP-68	^b^ND	0.090 ± 0.01	0.022 ± 0.078	0.14 ± 0.080	0.31 ± 0.95	0.18 ± 0.34	0.74
PMRP-69	0.028 ± 0.16	0.20 ± 0.56	0.080 ± 0.03	0.27 ± 0.42	1.36 ± 2.16	1.45 ± 4.49	3.41
PMRP-70	^b^ND	0.089 ± 0.01	0.022 ± 0.019	0.14 ± 0.00	0.48 ± 0.49	0.38 ± 0.40	1.11
PMRP-71	1.19 ± 4.47	0.85 ± 1.93	0.89 ± 3.43	0.81 ± 4.23	2.32 ± 18.15	2.21 ± 17.49	8.27
PMRP-72	0.40 ± 1.45	1.21 ± 4.70	0.50 ± 0.19	1.52 ± 2.72	6.52 ± 22.57	5.29 ± 18.23	15.44
PMRP-73	0.45 ± 0.29	0.78 ± 1.43	0.47 ± 0.79	1.21 ± 0.37	6.23 ± 10.35	5.98 ± 16.38	15.12
PMRP-74	0.054 ± 0.03	0.19 ± 0.53	0.059 ± 0.18	0.23 ± 0.39	3.41 ± 2.97	3.25 ± 2.33	7.19
PMRP-75	0.029 ± 0.14	1.25 ± 0.29	0.36 ± 0.24	1.71 ± 0.16	10.67 ± 0.60	10.10 ± 1.88	24.12
PMRP-76	0.55 ± 0.50	0.43 ± 0.20	0.45 ± 0.81	0.51 ± 0.51	1.20 ± 0.79	1.14 ± 0.16	4.28
PMRP-77	1.15 ± 1.12	0.97 ± 0.54	0.98 ± 2.83	1.00 ± 0.48	6.13 ± 11.15	5.36 ± 37.35	15.59
PMRP-78	1.18 ± 0.21	0.95 ± 3.74	1.03 ± 0.82	0.97 ± 1.22	6.23 ± 9.83	5.37 ± 15.03	15.73
PMRP-79	1.34 ± 0.75	1.12 ± 2.17	1.15 ± 1.72	1.08 ± 3.02	6.22 ± 5.22	5.48 ± 4.66	16.39
PMRP-80	1.30 ± 0.68	0.91 ± 8.23	0.90 ± 0.99	1.48 ± 1.62	1.41 ± 12.83	1.44 ± 3.38	7.44
PMRP-81	0.20 ± 0.39	0.47 ± 1.71	0.22 ± 0.93	0.73 ± 2.19	1.73 ± 1.46	1.44 ± 3.51	4.79
PMRP-82	^b^ND	0.090 ± 0.01	0.022 ± 0.081	0.14 ± 0.12	0.38 ± 0.60	0.27 ± 0.45	0.90
PMRP-83	0.19 ± 0.20	3.13 ± 1.39	1.84 ± 0.42	4.48 ± 1.85	19.83 ± 2.04	21.69 ± 1.97	51.16
PMRP-84	0.849 ± 1.01	1.03 ± 0.79	0.76 ± 1.06	1.73 ± 3.45	5.40 ± 12.61	5.86 ± 22.91	15.63
PMRP-85	0.269 ± 1.38	0.51 ± 0.96	0.26 ± 0.15	0.71 ± 1.08	1.38 ± 0.96	1.29 ± 0.34	4.42
PMRP-86	0.65 ± 1.37	6.80 ± 1.29	3.53 ± 0.35	9.55 ± 3.24	15.04 ± 3.08	15.83 ± 0.01	51.40
Average	0.96	1.08	0.71	1.53	4.47	4.25	12.99

^a^The data are presented as the average of two replicates

^b^ND, under limits of quantitation

^c^SD% is presented in the table

In above-mentioned experiments, the quality control (QC) samples consist of standard solutions of different concentrations and they were injected every 24 h. According to the literature [9], the best technology to process PMR was to steam for 24 h to eliminate the potential hepatotoxicity of PM. Further analysis was performed by focusing on the 45 samples of PMRP using the water-steaming method, since this processing technology is the most commonly used and has been recommended by the Chinese Pharmacopoeia. The contents of **5** and **6** decreased from 17.52 to 0.78 µg/g and 13.11 to 0.73 µg/g, respectively. The possible limit of the total contents of **5** and **6** could be no more than 1.51 µg/g in PMRP. If this possible limit is used to evaluate different PMRP samples on the market, more than 65% of the 86 commercial PMRP samples exceeded this limit. Therefore, it is noteworthy that there are problems with the processing methods of commercial PMRP.

#### Cytotoxicity evaluation of dianthrones in HepaRG cells

The two potentially toxic compounds, 5 and 6, were evaluated for their cytotoxicity in HepaRG cells by CCK-8 assay. According to the concentration-HepaRG cell inhibition rate curves drawn at different concentrations of the compounds, the IC_50_ values of each compound in the HepaRG cell model were determined. The IC_50_ values of compounds **5** and **6** were 5.60 μg/mL and 7.88 μg/mL, respectively. These values corresponded to 10.98 μM and 15.45 μM, respectively. The results suggested that compounds **5** and **6** had strong hepatocellular toxicity and could be used as potential toxicity markers.

## Discussion

**Fig. 5:**
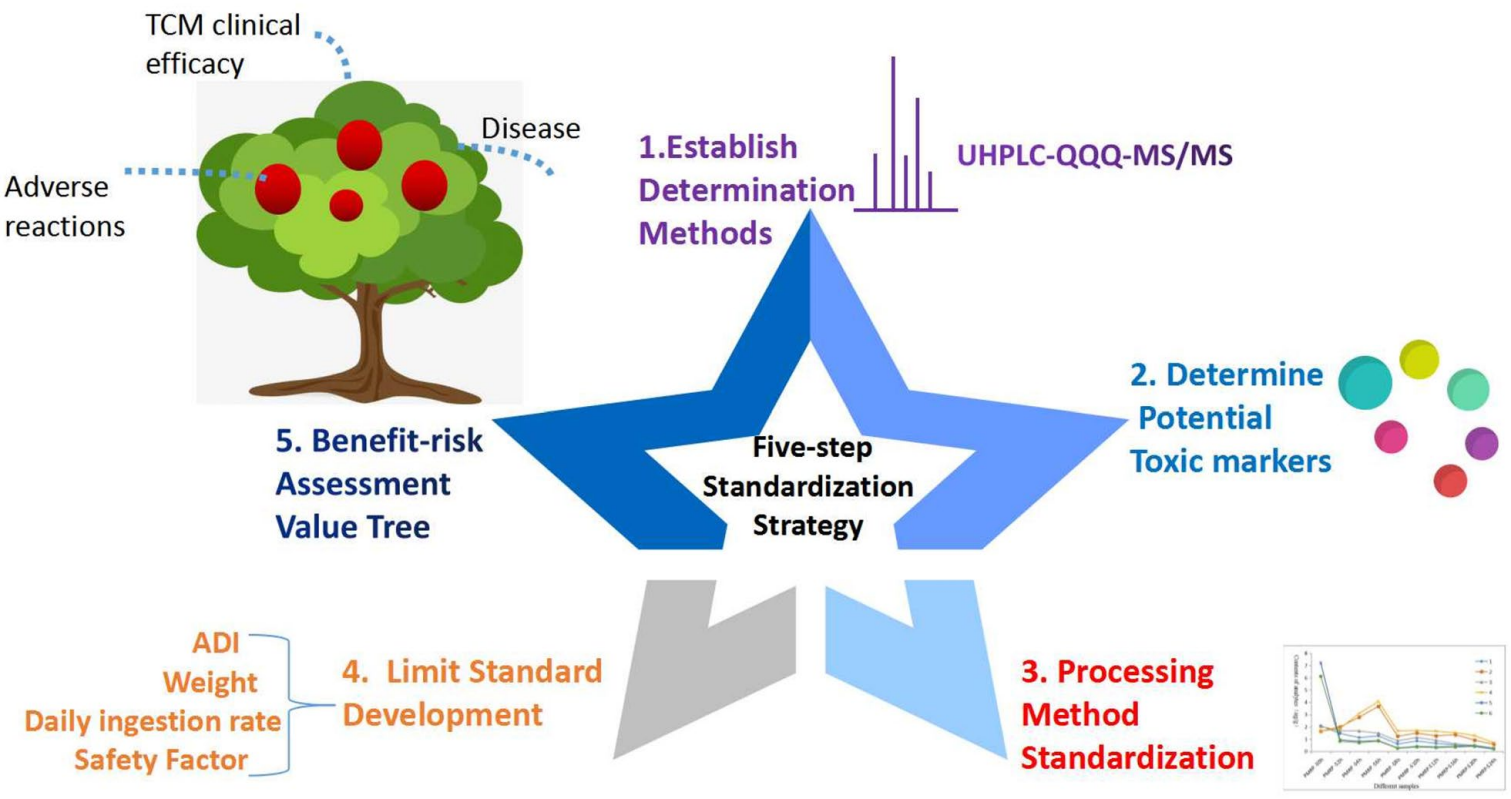
A systematic five-step strategy for standardization of endogenous toxic TCMs

TCMs with endogenous toxicity have a relatively narrow treatment window. If they are used improperly in the clinic, severe adverse reactions may occur. Attention has been directed towards TCMs with endogenous toxicity because of the negative effects and serious risks they cause to humans. Therefore, it is essential to develop a system to standardize TCMs with endogenous toxicity to guide the clinical use of TCMs. For the first time, in the present study, a systematic five-step strategy standardize TCMs with endogenous toxicity was proposed and involved the establishment of determination methods, the determination of toxic markers, the standardization of the processing method, the development of limit standards and a risk–benefit assessment (Fig. 5).

First, determination methods are expected to be developed to isolate and identify endogenous toxic chemicals in TCMs. The present study innovatively established a UHPLC-QQQ-MS/MS technique to simultaneously detect six dianthrones in PMR and PMRP. UHPLC-QQQ-MS/MS techniques are widely used for applications in chromatography–MS analysis. The method developed herein could not only provide rapid and improved chromatographic separation and a shorter chromatographic run time but can also provide higher sensitivity and selectivity, which are ultimately helpful for determining the contents of dianthrones in PMR and PMRP.

**Scheme 1:**
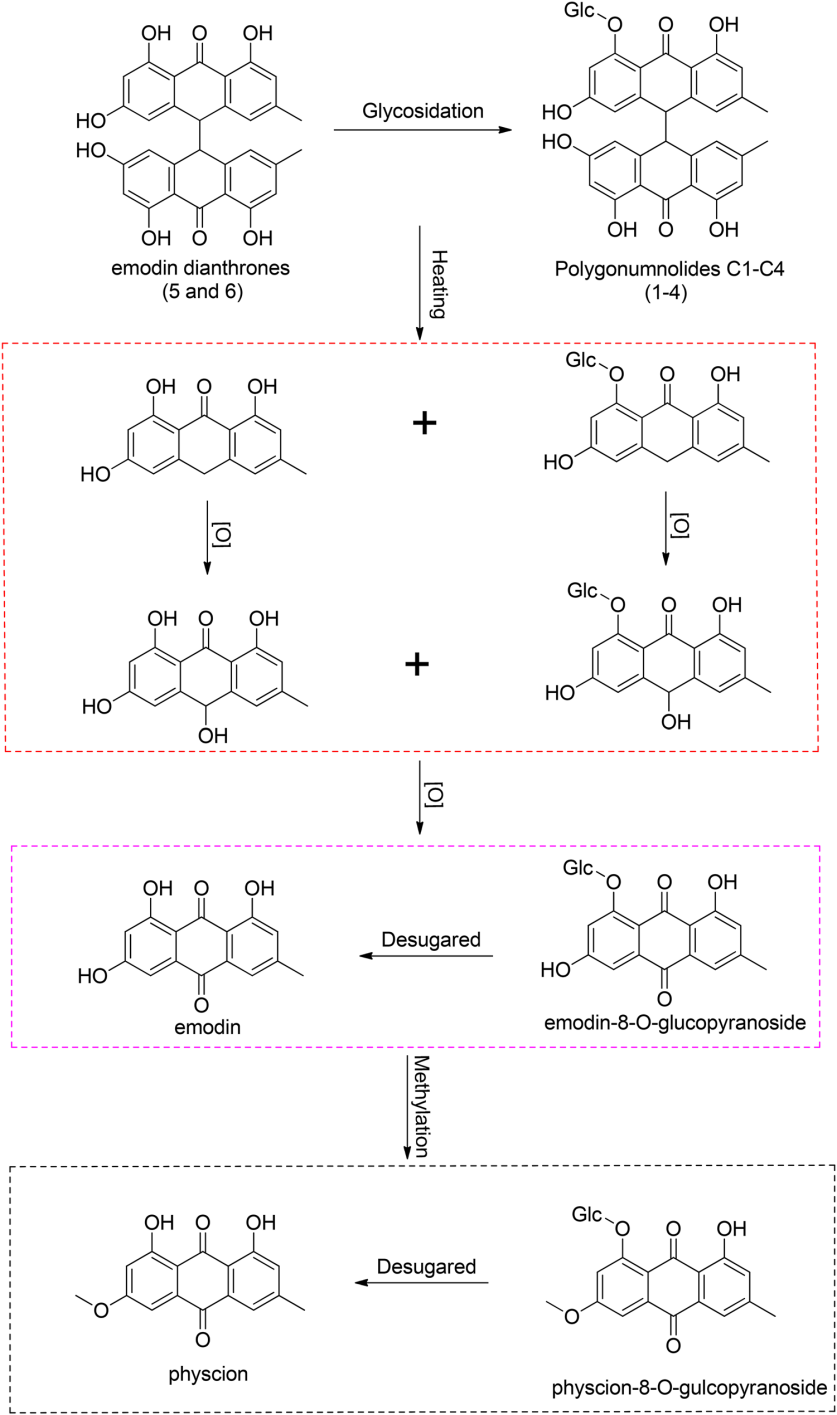
Hypothetical degradation process of dianthrones (**1**–**6**) in PMRP

Second, determining toxic markers and clarifying the mechanism could decrease the toxicity of TCMs. Interestingly, this study showed that dianthrones are widely distributed in PMR and demonstrated that these compounds, especially *trans*-emodin dianthrones (**5**) and *cis*-emodin dianthrones (**6**), could be selected as potential toxic markers of PMRP [11, 17]. The possible degradation process of the 6 dianthrones (**1**–**6**) in PMRP are as follows. Free dianthrones (**5** and **6**) may undergo glycosidation and be further converted into the combined dianthrones **1**–**4**. On the other hand, the C_10_–${\text{C}}_{{10}} ^{\prime }$ dianthrone bond could be easily cleaved under heating conditions. These dianthrones could be converted into anthrones and then further oxidized into anthracenols. Anthracenols may be further oxidized into anthraquinones, such as emodin and emodin-8-*O*-glucopyranoside, which may undergo methylation. Finally, the combined anthraquinones could be converted into free anthraquinones originating from the loss of the glucoside unit. Accordingly, the postulated degradation process of dianthrones in PMRP was speculated as Scheme 1. The contents of dianthrones may decrease significantly after reasonable processing. Therefore, this study could provide a theoretical basis to explore the mechanism of decreasing the toxicity of PMRP.

Third, standardization of the processing method is of great significance. Taking *P. multiflorum* preparations (PMPs) as an example, this study illustrated the relationship between different solvent extracts and the contents of dianthrones in PMRs and PMRPs for the first time. Different extracts using ethanol at different concentrations as an extracting agent could significantly influenced the hepatotoxicity of PMR, as reported in the references [28–30]. Therefore, five different concentrations of aqueous ethanol were chosen to evaluate the extraction efficiency of the dianthrones in this study. The results showed that 70% ethanol exhibited the highest extraction efficiency among the tested solvents. Interestingly, these results were consistent with our previous research, which also showed that the toxicity of the 70% ethanol extract was considered to be higher than that of other extracts, such as the H_2_O extract and 30% ethanol extract [11]. Furthermore, the present study showed that after extraction by the water-steaming method, the total contents of the 6 types of dianthrones decreased by more than 80%. Therefore, the extraction method of PMR is closely related to the contents of dianthrones. In addition, our study demonstrated that dianthrones were potential toxic markers of PMR, indicating that the extraction method is of significance for the potential toxicity of PMR and PMRP. Based on the results of this study and previous studies, it was suggested to pre-treat the PMR with the water-steaming method for 24 h. On the other hand, extraction with 70% ethanol is not encouraged. Overall, in the interest of public health, the standardization of pre-treatment methods is recommended to minimize the toxicity of TCMs with endogenous toxicity.

Fourth, considering public confidence in the safe use of TCMs and TCM preparations, the development of a scientific and practical limit standard for TCMs with endogenous toxicity is beneficial and urgently needed. Taking *P. multiflorum* preparations (PMPs) as an example, there are more than 300 Chinese patent medicines (CPMs) containing PMRs and PMRPs in the Chinese Pharmacopoeia and Drug Standard of the Ministry of Public Health of the People’s Republic of China [3, 31]. It has been reported that many PMPs, such as Yangxue Shengfa capsules, show certain hepatotoxicity [31–33]. However, to the best of our knowledge, there is no regulatory standard for PMR or PMP. Therefore, it is necessary to determine the limit standards for these dianthrones in PMR or PMP to guarantee medicinal safety of TCMs in the future. An appropriate method to formulate limit standards is the key. A scientific and practical limit standard should be based on the toxicological characteristics of the contained chemicals, the amount of TCM or TCM preparation ingested by the consumer, body weight, and safety factors. The following formula to calculate the maximum theoretical limit is recommended: *L* = *AWδ/M* (1) where L is the maximum theoretical limit, W is the body weight (70 kg), and M is the daily ingestion rate of the TCM or TCM preparation (g/day), which could be based on the consumption rate in the Pharmacopoeia of the People’s Republic of China (PPRC), and *δ* is a safety factor, accounting for the contribution of dietary supplements as a component of daily food intake. According to the judgement of the National Science Foundation (NSF), δ could be 10. A is the acceptable daily intake (ADI), which is defined as the estimated amount of a chemical to which a person can be exposed on a daily basis over a lifetime without suffering a detectable deleterious effect. For some endogenous toxic chemicals, such as pyrrolizidine alkaloids, ADI values have been set by organizations involving the World Health Organization (WHO) and European Food Safety Authority (EFSA), as references. However, for other endogenous toxic chemicals, such as the dianthrones in PMR or PMP, the ADI should be determined under the guidance of Good Laboratory Practice (GLP). In future studies, we will make great efforts to determine the crucial parameter ADI, especially the ADI for *trans*-emodin dianthrones (**5**) and *cis*-emodin dianthrones (**6**), based on which the maximum theoretical limit could be acquired. A practical maximum theoretical limit is the basis of a practical limit standard, and other factors involved in economic development, human cognition, and even history and culture, are recommended to be considered to maintain a balance between public safety and economic progress.

Finally, it is necessary to establish a benefit and risk assessment model of TCMs with endogenous toxicity to comprehensively evaluate the benefits and risks of TCMs and ensure both their safety and effectiveness. The evaluation of the risk–benefit ratio is determined by many factors and involves the establishment of a value tree of the risk–benefit ratio. The value tree is composed of the characteristics of the disease, clinical efficacy of the TCM, adverse reactions caused by the TCM, etc. On the basis of the severity, duration and incidence of the adverse reactions caused by TCMs with endogenous toxicity, these indexes should be weighed to obtain the estimated risk–benefit ratio. Moreover, it is paramount to build a very large mass spectral database to identify endogenous toxic chemicals, including dianthrones, as well as accumulate a wider range of extensive health risk assessment data on these endogenous toxic chemicals.

## Conclusions

In the present study, a rapid, sensitive, precise, and reliable UHPLC-QQQ-MS/MS method was developed for the simultaneous determination of six dianthrones in PMR and PMRP for the first time. The results indicated that *trans*-emodin dianthrones (**5**) and *cis*-emodin dianthrones (**6**) could be considered as potentially toxic markers of PMRP. Furthermore, taking PMR as an example, a systematic five-step strategy to promote the standardization of TCMs with endogenous toxicity was proposed for the first time, covering the research gap in this field. The systematic strategy consisted of the following steps: the establishment of determination methods, the identification of toxic markers, the standardization of the processing method, the development of limit standards and a risk–benefit assessment. Taking PMR and PMRP as examples, we hope this study provides insight into the standardization and internationalization of endogenous toxic TCMs and is conducive to improving the quality standard of these endogenous toxic TCMs and ensuring safe and effective clinical treatment.

## Data Availability

The research data generated from this study are included within the article.
